# Supplementary material for: Forced enhancer-promoter rewiring to alter gene expression in animal models
Source: Mol Ther Nucleic Acids. 2023 Jan 31;31:452–65. doi: 10.1016/j.omtn.2023.01.016 (PMC9958407; doi:10.1016/j.omtn.2023.01.016)
Supplement: Document S1. Figures S1–S7 and Tables S1–S4 [file mmc1.pdf]

OMTN, Volume *31*

## Supplemental information

# Forced enhancer-promoter rewiring

# to alter gene expression in animal models

**Scott A. Peslak, Selami Demirci, Vemika Chandra, Byoung Ryu, Saurabh K. Bhardwaj, Jing Jiang, Jeremy W. Rupon, Robert E. Throm, Naoya Uchida, Alexis Leonard, Khaled Essawi, Aylin C. Bonifacino, Allen E. Krouse, Nathaniel S. Linde, Robert E. Donahue, Francesca Ferrara, Matthew Wielgosz, Osheiza Abdulmalik, Nicole Hamagami, Paula Germino-Watnick, Anh Le, Rebecca Chu, Malikiya Hinds, Mitchell J. Weiss, Wei Tong, John F. Tisdale, and Gerd A. Blobel**

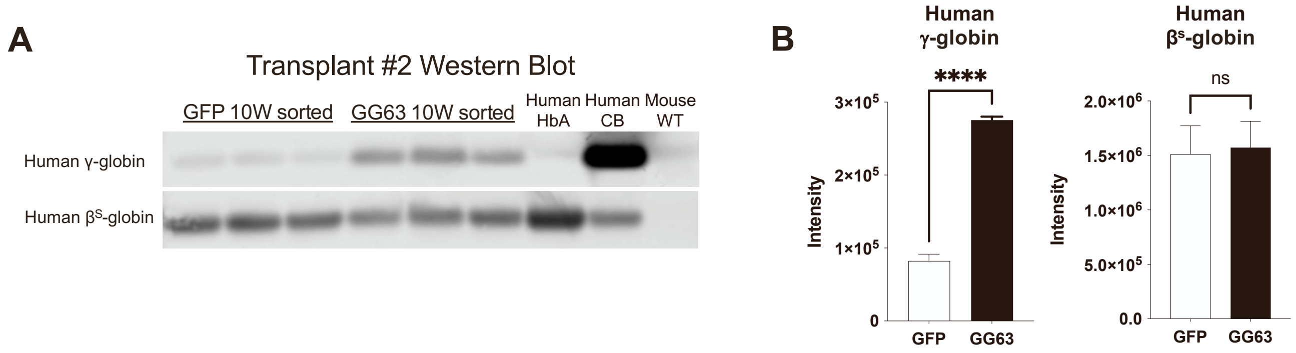


**Figure S1. Forced ZF-Ldb1 chromatin looping in the BERK mouse model drives in vivo induction of γ-globin expression.** (A) Western blot of an independent transplantation experiment utilizing the GG63-Ldb1-GFP construct showed consistent, sustained in vivo expression of γ-globin as compared to GFP control. (B) Quantification of γ-globin and $\beta^S$-globin proteins. N=3 transplanted mice for each condition. Statistical analysis done using Student's t-test. Error bars represent standard deviation; ns, not significant; ****, $p<0.0001$. CB, cord blood; WT, wild-type.

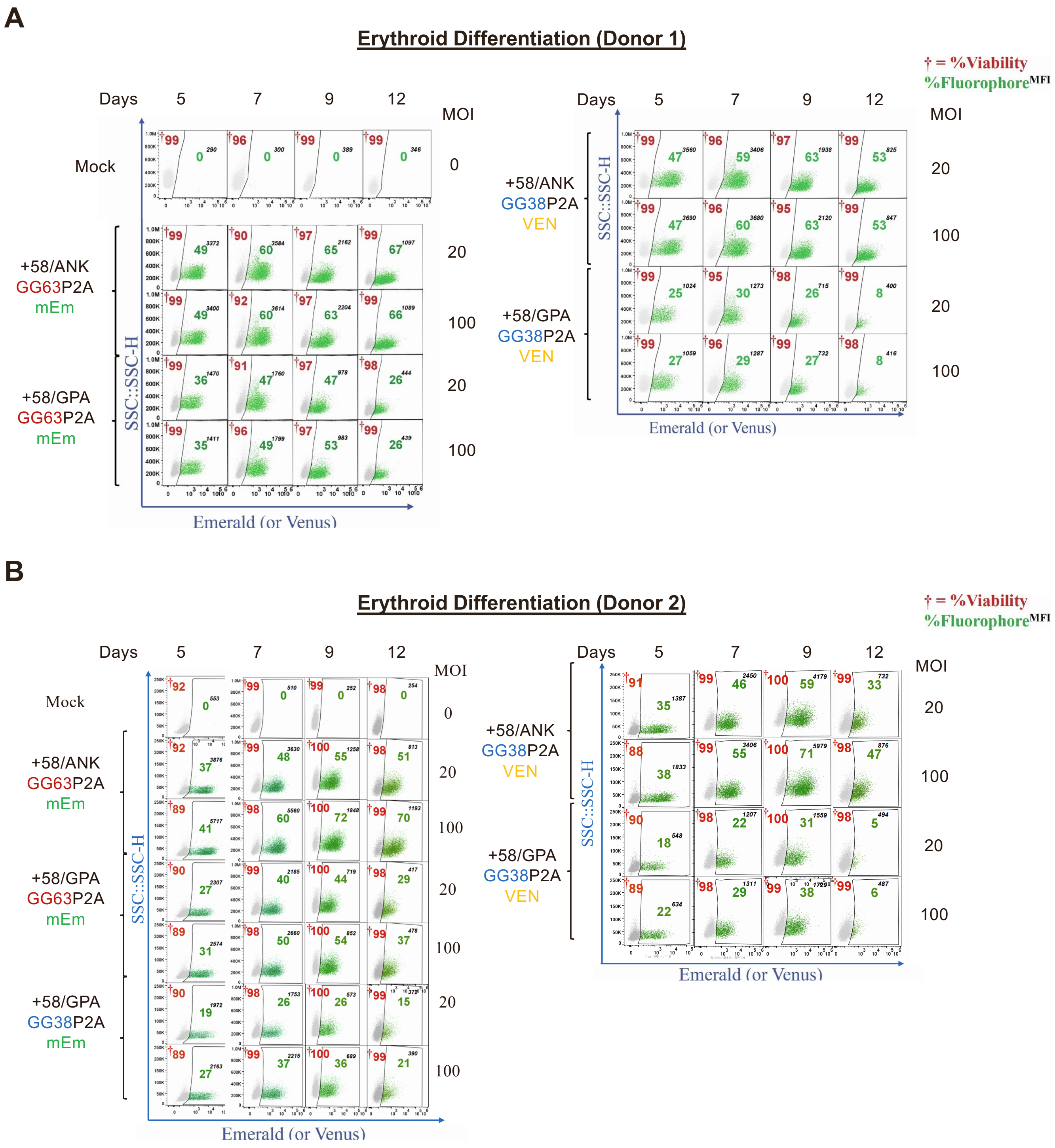


**Figure S2. Transgene expression and viability analysis of transduced rhesus CD34$^{+}$ cells.** Data shown at days 5, 7, 9, and 12 of in vitro erythroid culture for donor #1 (A) and donor #2 (B). Cellular viability is shown in red (% viability). Vector expression (measured by percent mEmerald or Venus by flow cytometric analysis) is quantified in green, with mean fluorescence intensity (MFI) as superscript (%Fluorophore$^{MFI}$). N=2 independent donors.

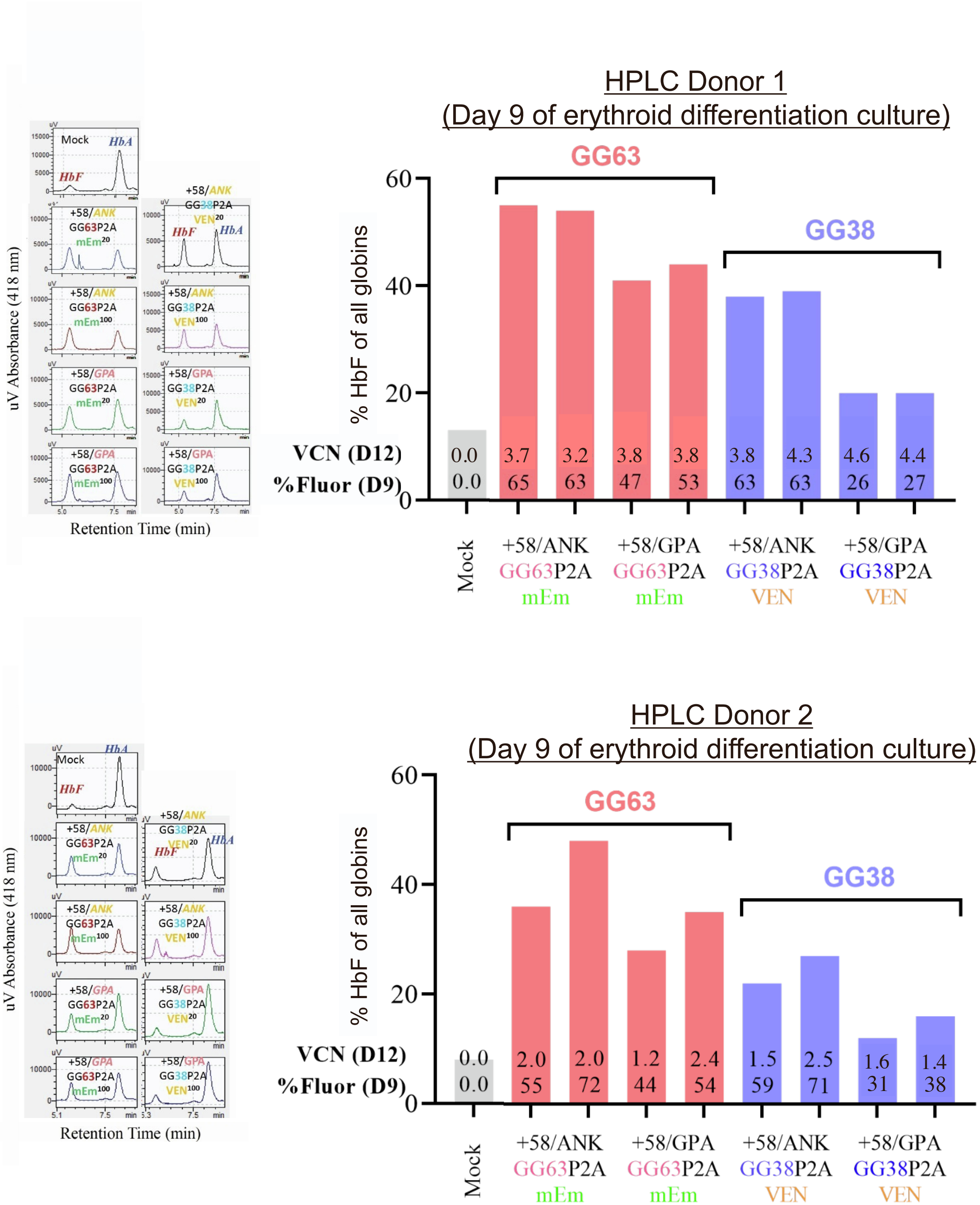


**Figure S3**. **HPLC analysis following transduction of optimized zinc finger constructs in two independent human CD34⁺ donor cells at day 9 of erythroid differentiation culture.** HPLC elution tracings (left) and quantification of HPLC peaks (right) illustrate high levels of HbF induction. Vector copy number (VCN D12) and percent Venus/mEmerald positivity (%Fluor D9) are displayed for each vector tested. N=2 independent donors.

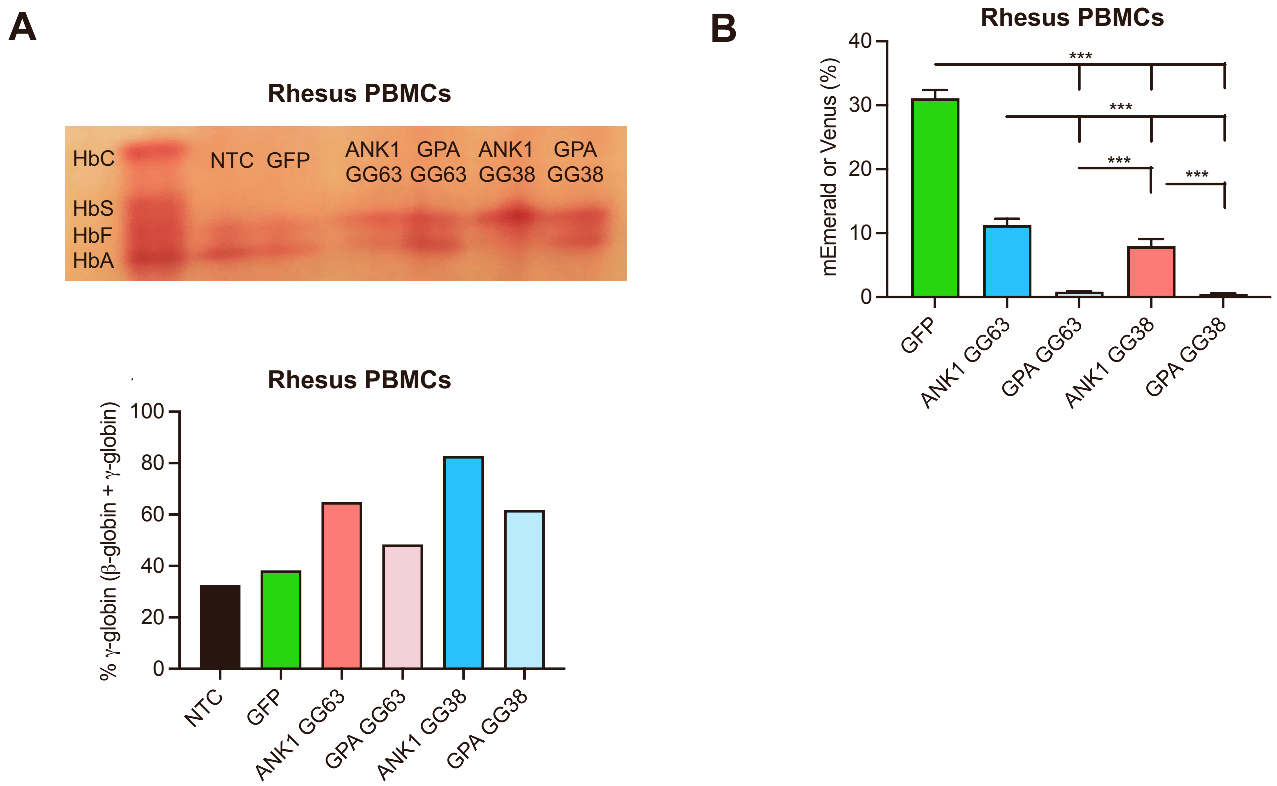


**Figure S4. Robust ex vivo γ-globin induction in rhesus progenitor cells transduced with optimized zinc finger constructs.** (A) Hemoglobin electrophoresis (top) and RP-HPLC (bottom) in differentiated rhesus PBMCs, N=1. (B) Flow cytometry analysis of mEmerald or Venus vector expression in differentiated rhesus PBMCs. Cells were transduced with the ZF lentiviruses (MOI=50 for $CD34^+$ and MOI=10 for PBMCs) at high cell density ($2\times10^6$cells/mL) in XVIVO-10 media + SFT (100 ng/mL each), N=3. Statistical analyses done using one-way ANOVA analysis. Error bars represent standard deviation; ***, $p<0.001$. HbF and flow cytometric assays were performed on unsorted bulk erythroid cells. HbC, hemoglobin C; HbS, hemoglobin S/sickle hemoglobin; HbF, hemoglobin F/fetal hemoglobin; HbA, hemoglobin A/adult hemoglobin.

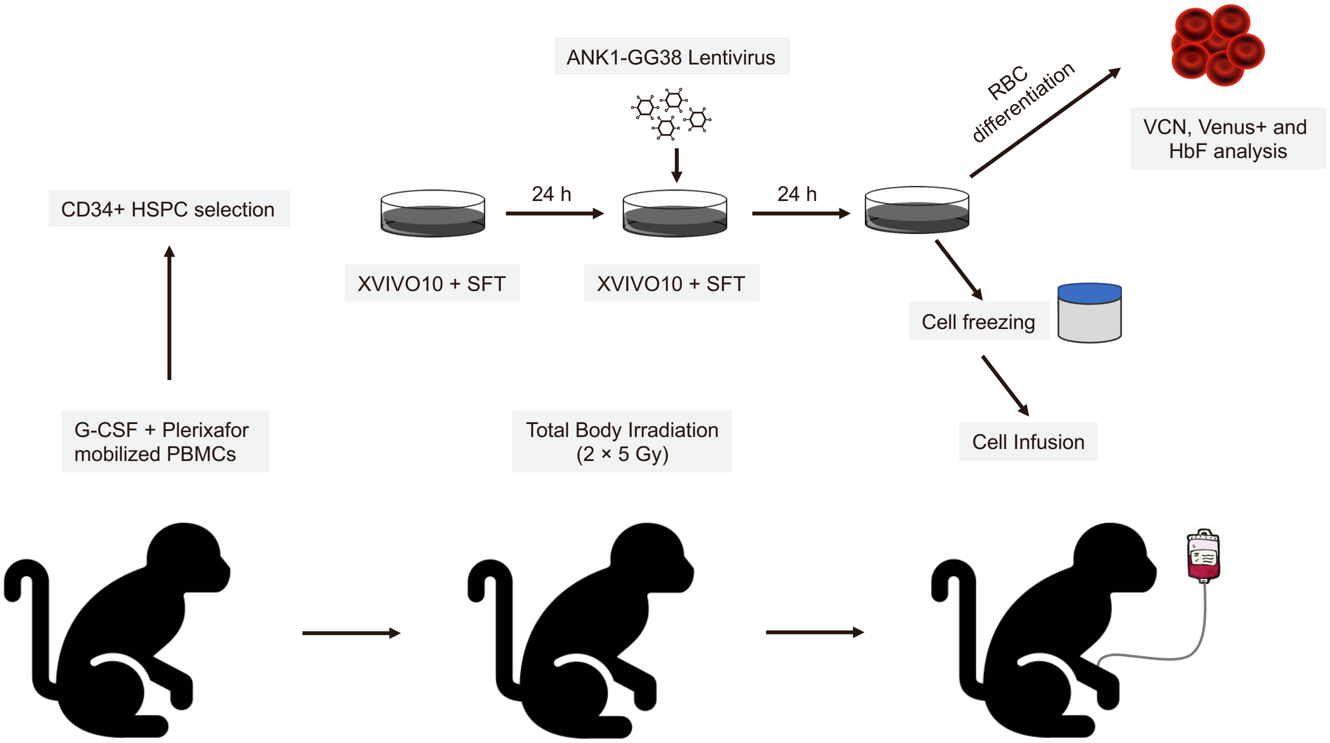


**Figure S5. Representative illustration for in vivo experimental design for rhesus macaque studies**. A small portion of the transduced $CD34^{+}$ hematopoietic stem and progenitor cells (HSPCs) were used for red blood cell (RBC) differentiation to confirm vector copy number (VCN), reporter gene (Venus), and fetal hemoglobin (HbF) induction before cell infusion. SFT, Stem cell factor, Flt-3, TPO.

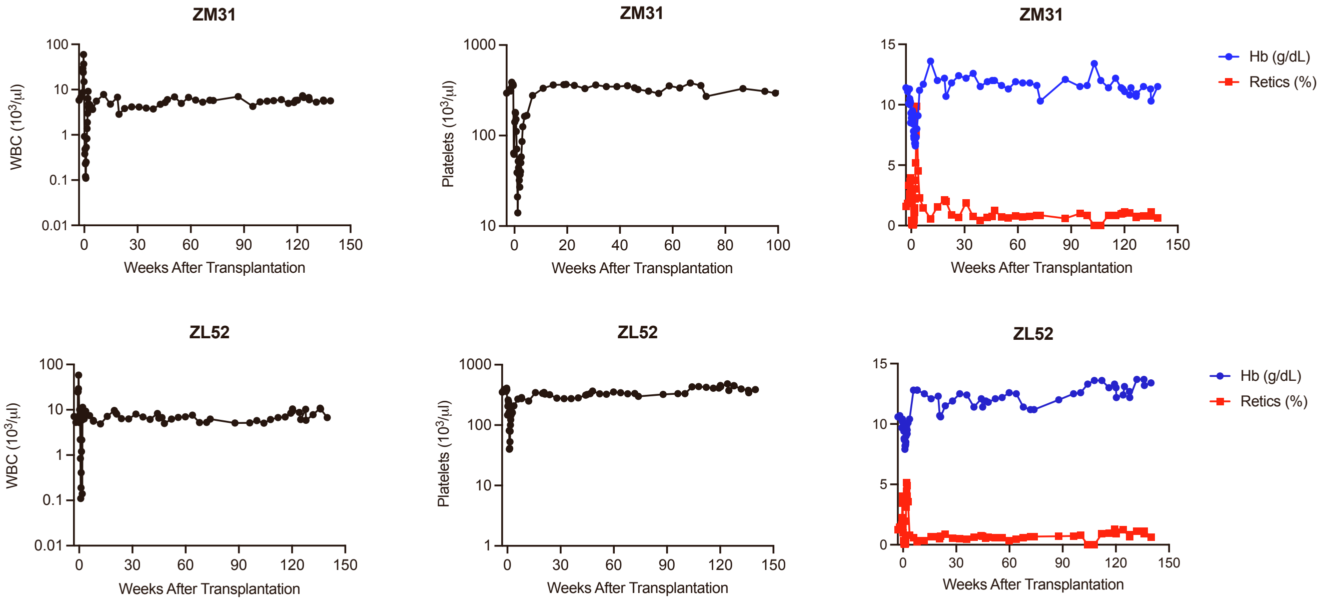


**Figure S6**. Peripheral blood parameters in transplanted rhesus macaques. WBC, white blood cells; Hb, hemoglobin; Retics, reticulocyte percentage. N=2 independent transplanted rhesus macaques.

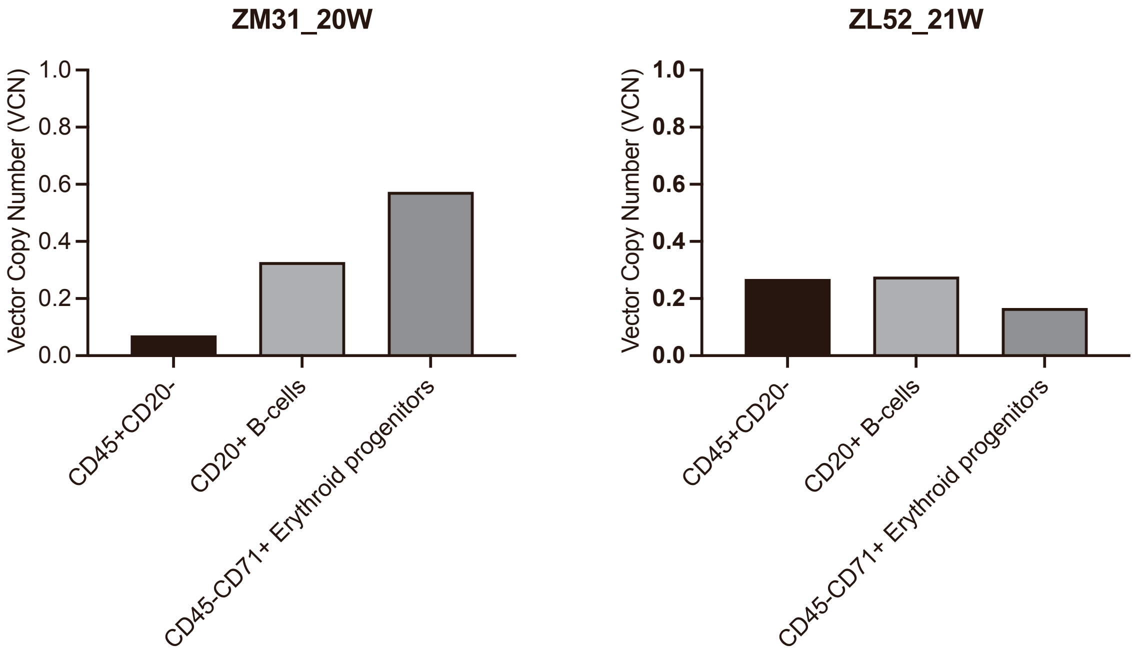


**Figure S7**. **Vector copy number analysis of transplanted macaques**. Vector copy number (VCN) was determined for $CD20^{+}$ B-cells, $CD45^{-}CD71^{+}$ erythroid progenitors, and all other $CD45^{+}$ cells ($CD45^{+}CD20^{-}$). N=2 independent transplanted rhesus macaques.

**Table S1. Differentially expressed genes (all DEG, N=33) by RNA-seq analysis following forced chromatin looping with GG38 ZF construct.** Differentially expressed genes (defined as absolute fold change greater than 1.5 and false discovery rate (FDR) less than 0.05) between GFP-only control and GG38 treated samples. Two biologically independent replicates were performed, and $\log_2$ fold change, p-value, and FDR are displayed for each differentially expressed gene.

| Gene Name | $\text{Log}_2$ Fold Change | p-value | FDR |
|---|---|---|---|
| **HBG2** | 3.391192224 | 1.03871850193996E-64 | 1.05637671647294E-61 |
| **HBG1** | 1.758872384 | 4.92265544578652E-18 | 2.50317029418245E-15 |
| **AC104389.6** | 1.887867842 | 3.68731774982421E-16 | 1.25000071719041E-13 |
| **RN7SL1** | 11.45843448 | 1.71914607021391E-14 | 4.37092888351887E-12 |
| **ENSG00000206028** | -2.015993142 | 7.40936805573879E-08 | 1.50706546253727E-05 |
| **CLK3** | -0.962098867 | 3.39970517514562E-07 | 5.76250027187182E-05 |
| **AC106788.3** | 2.308955752 | 7.94076631485196E-06 | 0.001150774 |
| **LMOD1** | 4.720262133 | 9.05230313845142E-06 | 0.001150774 |
| **HBD** | -1.244358975 | 1.68169128586907E-05 | 0.001900311 |
| **HBB** | -0.867365146 | 2.6758393268163E-05 | 0.002537381 |
| **ENSG00000265401** | -0.947692982 | 2.74446298830256E-05 | 0.002537381 |
| **PAQR3** | -0.849642798 | 4.00466594326636E-05 | 0.003393954 |
| **WDR5B-DT** | -0.972440856 | 5.22273379075885E-05 | 0.004085785 |
| **DDIT4-AS1:3** | 0.96084767 | 0.00010045 | 0.006816809 |
| **CCNDBP1** | -0.847377884 | 0.000100543 | 0.006816809 |
| **COG1** | -0.866773776 | 0.000156075 | 0.009920547 |
| **ENSG00000266642** | -0.793092242 | 0.000267332 | 0.015992741 |
| **PTOV1-AS1** | -1.131813167 | 0.000297157 | 0.016789377 |
| **RNF141** | -1.848561334 | 0.000344087 | 0.01841772 |
| **GMPPB** | -0.89928835 | 0.000497951 | 0.024385615 |
| **ENSG00000269984** | -1.023123598 | 0.000503538 | 0.024385615 |
| **ABALON** | -0.804037381 | 0.000690933 | 0.031627761 |
| **TAF10** | -0.943823154 | 0.000715279 | 0.031627761 |
| **IL16** | -1.402787456 | 0.000772927 | 0.03275279 |
| **ENSG00000279159** | -0.758754634 | 0.000819498 | 0.033337167 |
| **GADD45GIP1** | -0.738302076 | 0.000911435 | 0.035651132 |
| **STEAP3-AS1** | -1.067377965 | 0.001197563 | 0.042979078 |
| **TMEM50A** | -1.207911739 | 0.001214041 | 0.042979078 |
| **BEST3** | -0.929417047 | 0.001225559 | 0.042979078 |
| **LARP1** | -1.11365211 | 0.001333373 | 0.044527676 |
| **UBE2N** | -0.686687731 | 0.00139675 | 0.044527676 |
| **PSMG4** | -0.746330927 | 0.001401067 | 0.044527676 |
| **ENSG00000239665** | -1.009267904 | 0.001490096 | 0.04592205 |

**Table S2. Differentially expressed genes (all DEG, N=17) by RNA-seq analysis following forced chromatin looping with GG63 ZF construct.** Differentially expressed genes (defined as absolute fold change greater than 1.5 and false discovery rate (FDR) less than 0.05) between GFP-only control and GG63 treated samples. Two biologically independent replicates were performed, and $log_2$ fold change, p-value, and FDR are displayed for each differentially expressed gene.

| Gene Name | $Log_2$ Fold Change | p-value | FDR |
|---|---|---|---|
| HBG2 | 3.088062801 | 1.17832459239214E-66 | 5.19051982948736E-63 |
| HBG1 | 1.525015142 | 1.58389463268251E-15 | 3.48852792848322E-12 |
| AC104389.6 | 1.655987469 | 1.18598645747015E-13 | 1.74142344838533E-10 |
| HBB | -1.090908831 | 6.44017015221578E-09 | 7.09223738012763E-06 |
| IGF2 | 9.068903479 | 5.32468221730751E-08 | 4.41377700565219E-05 |
| ENSG00000260121 | 3.114699433 | 6.0119550587771E-08 | 4.41377700565219E-05 |
| ABLIM1 | 3.760551318 | 1.89172027210083E-07 | 0.000119043 |
| HBD | -1.842744945 | 1.21592899423866E-06 | 0.000669521 |
| ENSG00000262211 | -1.117812263 | 2.03091080037609E-06 | 0.000994018 |
| NUDT4B | 8.328044636 | 2.95126308678451E-06 | 0.001300031 |
| CLMN | 4.491272925 | 5.11289316647602E-06 | 0.002047481 |
| CLK3 | -0.668917158 | 5.09039212144048E-05 | 0.018685981 |
| GALNT7 | -1.272691299 | 5.899598873941E-05 | 0.019990564 |
| MSN | 4.763017708 | 9.58764175311498E-05 | 0.03016683 |
| GADD45GIP1 | -0.828050474 | 0.000140142 | 0.041154893 |
| ENSG00000266642 | -0.838953987 | 0.000152756 | 0.042055549 |
| CERKL | -0.800806786 | 0.000191864 | 0.04971524 |

**Table S3. Differentially expressed genes (top 100 by FDR, total DEG=409) by RNA-seq analysis following forced chromatin looping with GG52 ZF construct.** Differentially expressed genes (defined as absolute fold change greater than 1.5 and false discovery rate (FDR) less than 0.05) between GFP-only control and GG52 treated samples. Top 100 results sorted by FDR are displayed, total N=409 differentially expressed genes. Two biologically independent replicates were performed, and $log_2$ fold change, p-value, and FDR are displayed for each differentially expressed gene.

| Gene Name | $Log_2$ Fold Change | p-value | FDR |
|---|---|---|---|
| CLK3 | -2.432215887 | 5.14501390782645E-54 | 2.2056674622852E-50 |
| HBG2 | 2.794534378 | 1.19282894916777E-53 | 2.55682885254112E-50 |
| ENSG00000266642 | -2.094450993 | 1.31914672947036E-27 | 1.88506067641314E-24 |
| HEMGN | -1.985836414 | 8.42300079850547E-24 | 9.02735110579824E-21 |
| FAM149B1 | -1.479476299 | 7.12683537354198E-22 | 6.11054864927489E-19 |
| GMPPB | -1.986100799 | 2.54702507988224E-21 | 1.81984941957586E-18 |
| GGCX | 1.998808056 | 5.14827458097758E-19 | 3.15295044695013E-16 |
| HBG1 | 1.451239885 | 4.17923259898292E-17 | 2.23954626897997E-14 |
| ENSG00000272540 | -1.292472555 | 2.18213162352073E-16 | 1.0344070559669E-13 |
| FBXW8 | -2.862197616 | 2.41289259614392E-16 | 1.0344070559669E-13 |
| STEAP3-AS1 | -2.095233062 | 3.09379256095256E-14 | 1.16578653972603E-11 |
| CERKL | -1.393661481 | 3.26322334422962E-14 | 1.16578653972603E-11 |
| AC104389.6 | 1.618796832 | 6.90027640169904E-14 | 2.21502359841522E-11 |
| FTL | 1.759940212 | 7.23357368271825E-14 | 2.21502359841522E-11 |
| ENSG00000266872 | 2.905299274 | 9.01855388621453E-14 | 2.57750270068011E-11 |
| ENSG00000265401 | -1.362096235 | 3.08096303431498E-13 | 8.2550553300677E-11 |
| ENSG00000254680 | -2.142572587 | 4.71605508199926E-13 | 1.18927812567828E-10 |
| HK1 | -1.874265815 | 1.43178626081164E-12 | 3.2438332060107E-10 |
| ENSG00000247131 | -2.241311901 | 1.4376680875718E-12 | 3.2438332060107E-10 |
| SPATA9 | -1.323181237 | 2.39508977517318E-12 | 5.13387493308371E-10 |
| HSPA5 | 2.108172267 | 2.59831228391208E-12 | 5.30426893387196E-10 |
| PIGF | 5.08835732 | 5.31655915266376E-12 | 1.03600404943043E-09 |
| ENSG00000261684 | 1.440104194 | 7.17110285448898E-12 | 1.33663121466062E-09 |
| ENSG00000285103 | 2.897394706 | 8.45054599555291E-12 | 1.50947877845564E-09 |
| PRR29 | 3.000667646 | 9.73499132308371E-12 | 1.66935631208239E-09 |
| TMPO-AS1 | -1.791930918 | 1.21261674222299E-11 | 1.99941845150382E-09 |
| H2BC15 | -1.173938345 | 4.20710304703006E-11 | 6.67994472689551E-09 |
| HOOK2 | -2.373376925 | 5.24116429791466E-11 | 8.02459690898576E-09 |
| YIPF2 | -1.968112256 | 8.19931778021217E-11 | 1.21208535599205E-08 |
| TFRC | -1.226632033 | 9.86782669779182E-11 | 1.41011243511445E-08 |
| HSP90AB1 | 1.858908199 | 1.20811037890345E-10 | 1.67069974011583E-08 |
| IST1 | -1.420613869 | 1.27714718212911E-10 | 1.7109781155586E-08 |
| NEAT1 | 1.369457365 | 1.40791397251871E-10 | 1.82900824248113E-08 |
| S100A6 | 3.426210015 | 2.22037624904285E-10 | 2.79963322930785E-08 |
| ENSG00000265784 | 2.019372481 | 2.42630640843238E-10 | 2.97187873512846E-08 |
| LMNA | 2.122976882 | 3.20378179355004E-10 | 3.81517015248584E-08 |

| | | | |
|---|---|---|---|
| **COG1** | -1.211016937 | 3.42137678287137E-10 | 3.96417358599177E-08 |
| **ENSG00000258017** | -0.931803008 | 3.60162968815807E-10 | 4.06320696661412E-08 |
| **MAT2A** | 2.146230958 | 6.14654950633595E-10 | 6.75647634196467E-08 |
| **ENSG00000253389** | -1.325364487 | 1.72878339187919E-09 | 1.85282360024652E-07 |
| **NECAB1** | -1.149569167 | 2.26377261722549E-09 | 2.36702273415749E-07 |
| **GALNT7** | -1.383013007 | 3.23218369484442E-09 | 3.29913607138049E-07 |
| **BEST3** | -1.341359684 | 3.81775675245552E-09 | 3.8062146971574E-07 |
| **SIK2** | -1.608240528 | 4.6137811867478E-09 | 4.49529089717905E-07 |
| **APLNR** | 3.943399456 | 6.9390261547071E-09 | 6.61057891671763E-07 |
| **OR52A5** | -3.368108937 | 7.64987559924463E-09 | 7.12935145520907E-07 |
| **TMEM120B** | -3.350839786 | 1.03789975780793E-08 | 9.46697076962251E-07 |
| **PPP1R13B** | -1.651903258 | 1.06362219822436E-08 | 9.49947575789131E-07 |
| **ENSG00000279551** | -1.797627505 | 1.1477855907111E-08 | 1.00419527089357E-06 |
| **ENSG00000265263** | -1.230239236 | 1.43926389589559E-08 | 1.23402486434088E-06 |
| **ENSG00000267062** | -1.089797422 | 1.77932463680139E-08 | 1.49567935646423E-06 |
| **CAT** | -1.400720853 | 1.86997736100669E-08 | 1.54165248973763E-06 |
| **DNAJB12** | 2.341018477 | 2.38060116649923E-08 | 1.92559192467588E-06 |
| **ENSG00000262791** | -2.124481215 | 2.57242882587081E-08 | 2.04222266231633E-06 |
| **TSTD2** | -1.042460388 | 3.90932248060542E-08 | 3.04713917715554E-06 |
| **FARP1** | -0.87558818 | 4.03168041299383E-08 | 3.08639534473296E-06 |
| **MT-ND2** | 1.487581595 | 4.68346964350177E-08 | 3.52246216871791E-06 |
| **GYS1** | 2.643534403 | 5.45771125114104E-08 | 4.034001402352E-06 |
| **ENSG00000257607** | -1.304886578 | 5.90071146894155E-08 | 4.28751696056821E-06 |
| **ENSG00000280416** | 2.305103819 | 6.01264494482016E-08 | 4.296034813074E-06 |
| **ENSG00000285600** | -1.105966992 | 6.60233606843351E-08 | 4.64003520088106E-06 |
| **ERAL1** | -1.695640537 | 8.52063647077359E-08 | 5.89160783067845E-06 |
| **CLCC1** | -1.070144919 | 9.93627292182861E-08 | 6.76139714537766E-06 |
| **WDR73** | -1.361592893 | 1.02986321602645E-07 | 6.87985582652496E-06 |
| **DDIT4-AS1:3** | 0.967846836 | 1.04313186079805E-07 | 6.87985582652496E-06 |
| **PDP2** | 8.404999222 | 1.30309910688657E-07 | 8.46422101700411E-06 |
| **LCORL** | -1.133234731 | 1.34671793542554E-07 | 8.61698475995419E-06 |
| **INTS8** | -1.248248627 | 1.53055130336387E-07 | 9.56434196481623E-06 |
| **PRC1-AS1** | -1.161370769 | 1.53939723716426E-07 | 9.56434196481623E-06 |
| **STARD5** | -8.312250188 | 1.66914167694673E-07 | 1.02223005272437E-05 |
| **ENSG00000279159** | -0.936822329 | 2.01963173358758E-07 | 1.21945932984366E-05 |
| **MT-ATP6** | 1.74044102 | 2.07548089903493E-07 | 1.23577591863371E-05 |
| **OSBPL11** | 2.820540008 | 2.21771549596352E-07 | 1.30237620975282E-05 |
| **OR10Z1** | -0.861468838 | 2.3049770368742E-07 | 1.33532926447023E-05 |
| **STXBP5** | 3.898067589 | 2.40697126332593E-07 | 1.3758247741171E-05 |
| **CERS6-AS1** | 2.555660592 | 3.26838242995559E-07 | 1.84362572068679E-05 |
| **ENSG00000251600** | -1.266984569 | 4.43471804704887E-07 | 2.46904367112968E-05 |
| **GADD45GIP1** | -1.089370909 | 4.59576273954568E-07 | 2.52590190569645E-05 |
| **ENSG00000272418** | -1.752388205 | 4.98588668527815E-07 | 2.70563243288449E-05 |
| **AGK** | 2.62455101 | 5.30862067877239E-07 | 2.84475710623715E-05 |
| **ENSG00000282542** | -1.14572389 | 5.61956240855607E-07 | 2.97420543771356E-05 |
| **BTG1-DT** | 1.281131349 | 7.421411416597E-07 | 3.87995009060382E-05 |
| **ENSG00000238273** | -8.030862939 | 7.66442316030688E-07 | 3.95872073352236E-05 |
| **PLEKHG3** | -1.396937984 | 7.95494497679704E-07 | 4.05986298994392E-05 |
| **FRMD5** | -1.886777857 | 8.12017238489247E-07 | 4.09543282518047E-05 |
| **MALAT1** | 0.743623075 | 8.42079478056495E-07 | 4.19766828189325E-05 |

| | | | |
|---|---|---|---|
| **ATG14** | -1.444069684 | 8.62482221250726E-07 | 4.24995549712858E-05 |
| **ENSG00000228748** | 1.911656255 | 8.85223945913177E-07 | 4.31244892742022E-05 |
| **ARID3B** | -2.097838188 | 9.7829394788114E-07 | 4.71229905007466E-05 |
| **HBB** | -0.886178508 | 1.0075573767192E-06 | 4.79933163777246E-05 |
| **MAP3K4** | 4.294729186 | 1.02649813700001E-06 | 4.83582144320772E-05 |
| **MIR1302-9HG** | -1.109902288 | 1.14525584150282E-06 | 5.33664325274194E-05 |
| **ABI3** | -2.815626944 | 1.29457789036755E-06 | 5.9675864688233E-05 |
| **ENSG00000239665** | -1.145742043 | 1.58733311890088E-06 | 7.2392522135405E-05 |
| **LMOD1** | 4.411125545 | 1.62811880884782E-06 | 7.34330618341621E-05 |
| **DIS3** | -1.136237265 | 1.64440726290636E-06 | 7.34330618341621E-05 |
| **TAF10** | -1.288007722 | 1.67234935615673E-06 | 7.39109452561226E-05 |
| **ENSG00000259248** | 1.465045418 | 1.70662532688518E-06 | 7.46561507791507E-05 |
| **ANXA1** | 7.26581351 | 1.74639463428412E-06 | 7.56241797694547E-05 |
| **ENSG00000206028** | -2.545660438 | 1.80122406151495E-06 | 7.72184755171457E-05 |

**Table S4. Differentially expressed genes (top 100 by FDR, total DEG=677) by RNA-seq analysis following forced chromatin looping with GG1 ZF construct.** Differentially expressed genes (defined as absolute fold change greater than 1.5 and false discovery rate (FDR) less than 0.05) between GFP-only control and GG1 treated samples. Top 100 results sorted by adjusted P value are displayed, total N=677 differentially expressed genes. Two biologically independent replicates were performed, and $log_2$ fold change, p-value, and FDR are displayed for each differentially expressed gene.

| Gene Name | $Log_2$ Fold Change | p-value | FDR |
|---|---|---|---|
| CLK3 | -2.727882 | 4.33237E-62 | 3.9078E-58 |
| ENSG00000206028 | -6.6416247 | 1.10015E-50 | 4.96168E-47 |
| HBG2 | 2.89445347 | 1.74479E-49 | 5.24601E-46 |
| CERKL | -2.664109 | 1.88434E-46 | 4.24918E-43 |
| ENSG00000262211 | -2.6360942 | 3.22787E-32 | 5.82308E-29 |
| HBD | -2.8674076 | 3.40427E-31 | 5.11776E-28 |
| STEAP3-AS1 | -3.4032636 | 4.89392E-31 | 6.30616E-28 |
| FAM149B1 | -1.748942 | 5.3688E-27 | 6.05332E-24 |
| GGCX | 2.28056268 | 4.16063E-26 | 4.16987E-23 |
| TCHP | 2.90312058 | 2.94641E-25 | 2.65766E-22 |
| ENSG00000269926 | 1.74724404 | 1.85639E-22 | 1.52224E-19 |
| ENSG00000265401 | -2.017093 | 6.07293E-22 | 4.56482E-19 |
| FAM83A-AS1 | 3.03841883 | 1.49474E-21 | 1.03712E-18 |
| TKT | 4.02062249 | 3.21024E-19 | 2.06831E-16 |
| HBG1 | 1.52098425 | 3.63148E-19 | 2.18373E-16 |
| BEST3 | -2.0061645 | 7.0176E-18 | 3.95617E-15 |
| YIPF2 | -2.1085721 | 1.00445E-16 | 5.32948E-14 |
| ENSG00000251095 | -2.2396276 | 1.36542E-16 | 6.8423E-14 |
| GMPPB | -1.6288886 | 2.45552E-16 | 1.16573E-13 |
| ENSG00000247324 | -3.3226552 | 2.40442E-15 | 1.08439E-12 |
| IST1 | -1.6858456 | 5.63388E-15 | 2.41988E-12 |
| FAM13A-AS1 | -2.7404672 | 8.14426E-15 | 3.33914E-12 |
| ENSG00000247131 | -2.3657665 | 2.05591E-14 | 8.06275E-12 |
| SNCA | -2.6385337 | 3.59215E-14 | 1.35005E-11 |
| DMAC2L | -1.9300216 | 4.8047E-14 | 1.73354E-11 |
| FAM129A | 1.84287041 | 2.58197E-13 | 8.95745E-11 |
| CCDC144CP | 2.9088118 | 4.0613E-13 | 1.31888E-10 |
| MAT2A | 2.33435158 | 4.09408E-13 | 1.31888E-10 |
| TAX1BP1 | -1.6173907 | 7.57117E-13 | 2.35489E-10 |
| JMJD1C | -2.1215031 | 9.3127E-13 | 2.80002E-10 |
| TMCC2 | -2.4710061 | 1.1074E-12 | 3.22218E-10 |
| ENSG00000280064 | 2.12617256 | 1.15923E-12 | 3.26757E-10 |
| UBB | -1.409654 | 1.38897E-12 | 3.69512E-10 |
| RNF141 | -2.7877697 | 1.39284E-12 | 3.69512E-10 |
| PPP2R5B | 2.66671694 | 2.05689E-12 | 5.30089E-10 |
| PPME1 | -3.2430808 | 3.19299E-12 | 8.00022E-10 |

| | | | |
|---|---|---|---|
| **AC104389.6** | 1.51765529 | 3.49603E-12 | 8.28771E-10 |
| **NUDT4** | 1.15551401 | 3.50013E-12 | 8.28771E-10 |
| **XPOT** | 1.53566636 | 3.58374E-12 | 8.28771E-10 |
| **ENSG00000272418** | -3.0951656 | 3.67526E-12 | 8.28771E-10 |
| **ENSG00000285830** | 1.34729276 | 7.92642E-12 | 1.74381E-09 |
| **C12orf60** | -2.5189727 | 8.66542E-12 | 1.861E-09 |
| **CAT** | -1.7437091 | 1.05152E-11 | 2.20575E-09 |
| **EEF1A1** | 1.4665236 | 1.15936E-11 | 2.37669E-09 |
| **SEC62** | -1.8334015 | 1.77299E-11 | 3.55387E-09 |
| **SMNDC1** | -1.4396419 | 1.85724E-11 | 3.64181E-09 |
| **BNIP3L** | -1.6708491 | 2.29132E-11 | 4.39738E-09 |
| **ENSG00000265263** | -1.6289464 | 3.76862E-11 | 7.08187E-09 |
| **CCDC152** | -2.7812199 | 4.09095E-11 | 7.53069E-09 |
| **ENSG00000269982** | -1.7087575 | 4.82142E-11 | 8.69785E-09 |
| **GRAP2** | 2.34138058 | 5.99169E-11 | 1.05971E-08 |
| **IDH1** | 3.22539098 | 9.49834E-11 | 1.6476E-08 |
| **STOM** | -1.4079948 | 1.59641E-10 | 2.7169E-08 |
| **DAPK1** | -2.3188293 | 1.8968E-10 | 3.16835E-08 |
| **CDC27** | -1.8985189 | 2.21937E-10 | 3.63977E-08 |
| **CNOT7** | 1.23001762 | 5.0876E-10 | 8.19467E-08 |
| **OR10Z1** | -1.1966461 | 5.42622E-10 | 8.58675E-08 |
| **SPATA9** | -1.1034353 | 5.78303E-10 | 8.99362E-08 |
| **SLC24A1** | -1.3000934 | 8.31217E-10 | 1.27078E-07 |
| **TEX2** | 1.50548989 | 9.40293E-10 | 1.41357E-07 |
| **TCP11L2** | -2.0383232 | 9.91944E-10 | 1.46678E-07 |
| **MCM5** | 1.18860682 | 1.19326E-09 | 1.73599E-07 |
| **RANBP10** | -2.8680447 | 1.22913E-09 | 1.7598E-07 |
| **C9orf78** | -2.2511255 | 2.06738E-09 | 2.91371E-07 |
| **CLMN** | 4.48172922 | 2.73087E-09 | 3.7896E-07 |
| **SMG1P3** | 2.45177729 | 2.83321E-09 | 3.87205E-07 |
| **HEMGN** | -1.2623691 | 3.11111E-09 | 4.18839E-07 |
| **MBNL1-AS1** | -2.6500498 | 4.15617E-09 | 5.51303E-07 |
| **ENSG00000175182** | -2.1569884 | 4.32653E-09 | 5.59361E-07 |
| **ENSG00000272211** | -2.0375892 | 4.36818E-09 | 5.59361E-07 |
| **ENSG00000279551** | -1.925053 | 4.40295E-09 | 5.59361E-07 |
| **ENSG00000253194** | -1.5993282 | 4.81322E-09 | 6.0299E-07 |
| **MOSPD1** | -3.7058273 | 5.18883E-09 | 6.4114E-07 |
| **PIM1** | -1.2379079 | 5.41846E-09 | 6.60466E-07 |
| **ENSG00000284669** | -1.6922905 | 6.31796E-09 | 7.5984E-07 |
| **NUDT4P2** | 1.54275152 | 7.35102E-09 | 8.7245E-07 |
| **ARSG** | -1.358277 | 8.72563E-09 | 1.02068E-06 |
| **CCDC144A** | 2.23651906 | 8.82626E-09 | 1.02068E-06 |
| **RAP1GAP** | -2.6962696 | 1.0246E-08 | 1.16987E-06 |
| **CHST2** | -4.8768465 | 1.05381E-08 | 1.18817E-06 |
| **COG1** | -1.0723265 | 1.15895E-08 | 1.29059E-06 |
| **SLC19A1** | -1.4439612 | 1.67746E-08 | 1.84521E-06 |
| **AGTR1** | -4.4141522 | 1.92568E-08 | 2.09273E-06 |
| **HSPA5** | 1.69708221 | 2.14206E-08 | 2.30016E-06 |
| **PDE4DIP** | 5.15618708 | 2.31852E-08 | 2.46036E-06 |
| **ARID3B** | -2.3297984 | 2.37443E-08 | 2.4904E-06 |

| | | | |
|---|---|---|---|
| **WARS1** | 2.07828994 | 2.63523E-08 | 2.70421E-06 |
| **ENSG00000269984** | -1.0989837 | 2.63825E-08 | 2.70421E-06 |
| **ACP1** | -3.099081 | 3.09075E-08 | 3.13242E-06 |
| **TENT5C** | -1.2195707 | 3.57755E-08 | 3.5855E-06 |
| **GLRX5** | -1.2682948 | 3.70601E-08 | 3.67343E-06 |
| **ENSG00000215859** | -3.2720296 | 6.43585E-08 | 6.30994E-06 |
| **SENP6** | 2.55161254 | 6.821E-08 | 6.61563E-06 |
| **TBCEL** | -2.1755542 | 6.97417E-08 | 6.69224E-06 |
| **PLIN2** | 2.14150985 | 7.47679E-08 | 7.09901E-06 |
| **CYREN** | -1.3391296 | 7.59953E-08 | 7.14039E-06 |
| **ALDH6A1** | -2.9337208 | 9.9102E-08 | 9.21546E-06 |
| **ENSG00000213742** | 1.09860857 | 1.01315E-07 | 9.32511E-06 |
| **INTS8** | -1.1800534 | 1.02525E-07 | 9.34118E-06 |
| **ENSG00000241570** | -1.3116248 | 1.05543E-07 | 9.51996E-06 |
