# Supplementary material for: Forced enhancer-promoter rewiring to alter gene expression in animal models
Source: Mol Ther Nucleic Acids. 2023 Jan 31;31:452–65. doi: 10.1016/j.omtn.2023.01.016 (PMC9958407; doi:10.1016/j.omtn.2023.01.016)
Supplement: Document S2. Article plus supplemental information [file mmc2.pdf]

Molecular Therapy
Nucleic Acids
Original Article

Forced enhancer-promoter rewiring to alter gene expression in animal models

Scott A. Peslak,[1,2,9] Selami Demirci,[3,9] Vemika Chandra,[2] Byoung Ryu,[4] Saurabh K. Bhardwaj,[2] Jing Jiang,[2,5] Jeremy W. Rupon,[2] Robert E. Throm,[4] Naoya Uchida,[3,6] Alexis Leonard,[3] Khaled Essawi,[3,7] Aylin C. Bonifacino,[8] Allen E. Krouse,[8] Nathaniel S. Linde,[8] Robert E. Donahue,[3] Francesca Ferrara,[4] Matthew Wielgosz,[4] Osheiza Abdulmalik,[2] Nicole Hamagami,[2] Paula Germino-Watnick,[3] Anh Le,[3] Rebecca Chu,[3] Malikiya Hinds,[3] Mitchell J. Weiss,[4] Wei Tong,[2] John F. Tisdale,[3] and Gerd A. Blobel[2]

[1]Division of Hematology/Oncology, Department of Medicine, University of Pennsylvania Perelman School of Medicine, Philadelphia, PA 19104, USA; [2]Division of Hematology, The Children's Hospital of Philadelphia, Philadelphia, PA 19104, USA; [3]Cellular and Molecular Therapeutics Branch, National Heart, Lung, and Blood Institutes (NHLBI), National Institutes of Health (NIH), Bethesda, MD 20892, USA; [4]Department of Hematology, St. Jude Children's Research Hospital, Memphis, TN 38105, USA; [5]CAS Engineering Laboratory for Nanozyme, Institute of Biophysics, Chinese Academy of Sciences, Beijing, People's Republic of China; [6]Division of Molecular and Medical Genetics, Center for Gene and Cell Therapy, The Institute of Medical Science, The University of Tokyo, Minato-ku, Tokyo, Japan; [7]Department of Medical Laboratory Science, College of Applied Medical Sciences, Jazan University, Jazan, Saudi Arabia; [8]Translational Stem Cell Biology Branch, NHLBI, NIH, Bethesda, MD 20814, USA

Transcriptional enhancers can be in physical proximity of their target genes via chromatin looping. The enhancer at the β-globin locus (locus control region [LCR]) contacts the fetal-type (*HBG*) and adult-type (*HBB*) β-globin genes during corresponding developmental stages. We have demonstrated previously that forcing proximity between the LCR and *HBG* genes in cultured adult-stage erythroid cells can activate *HBG* transcription. Activation of *HBG* expression in erythroid cells is of benefit to patients with sickle cell disease. Here, using the β-globin locus as a model, we provide proof of concept at the organismal level that forced enhancer rewiring might present a strategy to alter gene expression for therapeutic purposes. Hematopoietic stem and progenitor cells (HSPCs) from mice bearing human β-globin genes were transduced with lentiviral vectors expressing a synthetic transcription factor (ZF-Ldb1) that fosters LCR-*HBG* contacts. When engrafted into host animals, HSPCs gave rise to adult-type erythroid cells with elevated *HBG* expression. Vectors containing ZF-Ldb1 were optimized for activity in cultured human and rhesus macaque erythroid cells. Upon transplantation into rhesus macaques, erythroid cells from HSPCs expressing ZF-Ldb1 displayed elevated *HBG* production. These findings in two animal models suggest that forced redirection of gene-regulatory elements may be used to alter gene expression to treat disease.

INTRODUCTION

Transcriptional enhancers can be located at great genomic distances from their target promoters. Chromatin looping that physically juxtaposes enhancers and promoters is one mechanism by which regulatory information may be transmitted, even though there is a vigorous debate about how widespread this mechanism is and how "proximity" translates into actual physical distances.[1–4] The β-globin gene cluster comprises a distal enhancer, termed locus control region (LCR), that is required for high-level transcription of all β-type globin genes in erythroid cells.[5,6] Based on chromosome conformation capture (3C) experiments, the LCR is thought to contact globin genes[7,8] requiring erythroid-specific transcription factors,[9] including *GATA1* and its coregulators *FOG1* and Ldb1.[10,11] "Contact" is operationally defined here as an increased 3C signal with no implications for actual Euclidean distance.

LCR-gene contacts occur in a developmental-stage-specific manner. Thus, the LCR is in proximity with the fetal-type (*HBG1/2*) and adult-type (*HBB* and *HBD*) globin genes during fetal and adult stages, respectively.[12,13] Interest in the molecular underpinnings of the switch from *HBG* to *HBB* expression, which occurs around the time of birth, has been stimulated by the recognition that elevated fetal hemoglobin (hemoglobin F [HbF]) levels benefit patients with sickle cell disease (SCD), a devastating disorder caused by a point mutation affecting the *HBB* gene (20A>T, Glu6Val).[14–16] HbF consists of the products of the *HBG* and *HBA* genes ($\gamma_2\,\alpha_2$), while adult hemoglobin consists predominantly of the products of the *HBB* and *HBA* genes ($\beta_2\,\alpha_2$).

Our prior work examined the cause-effect relationship of looped enhancer-promoter contacts by forcing LCR-promoter interactions

Received 20 June 2022; accepted 25 January 2023;
https://doi.org/10.1016/j.omtn.2023.01.016.

[9]These authors contributed equally

Correspondence: John F. Tisdale, Cellular and Molecular Therapeutics Branch, National Heart, Lung, and Blood Institutes (NHLBI), National Institutes of Health (NIH), Bethesda, MD 20892, USA.
E-mail: johntis@nhlbi.nih.gov
Correspondence: Gerd A. Blobel, Division of Hematology, The Children's Hospital of Philadelphia, Philadelphia, PA 19104, USA.
E-mail: blobel@chop.edu

This is an open access article under the CC BY license (http://creativecommons.org/licenses/by/4.0/).

A

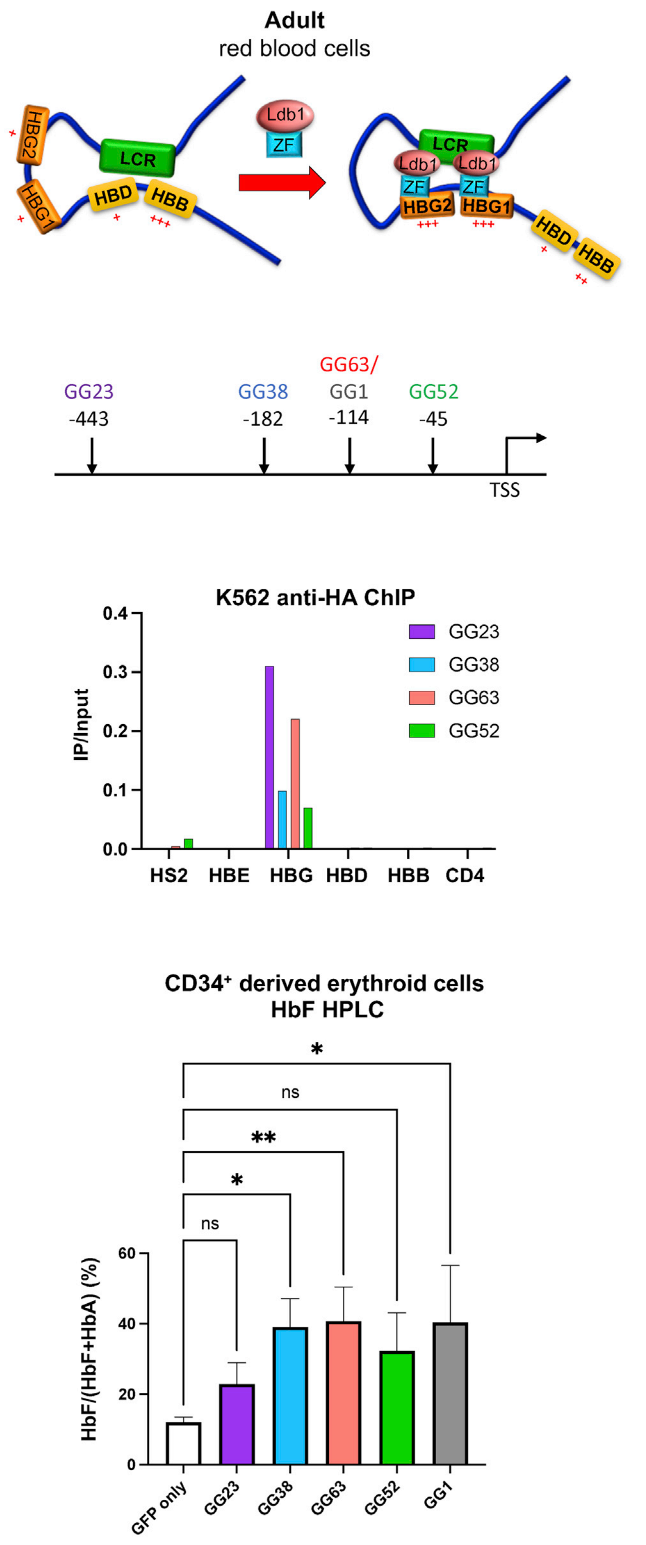

E

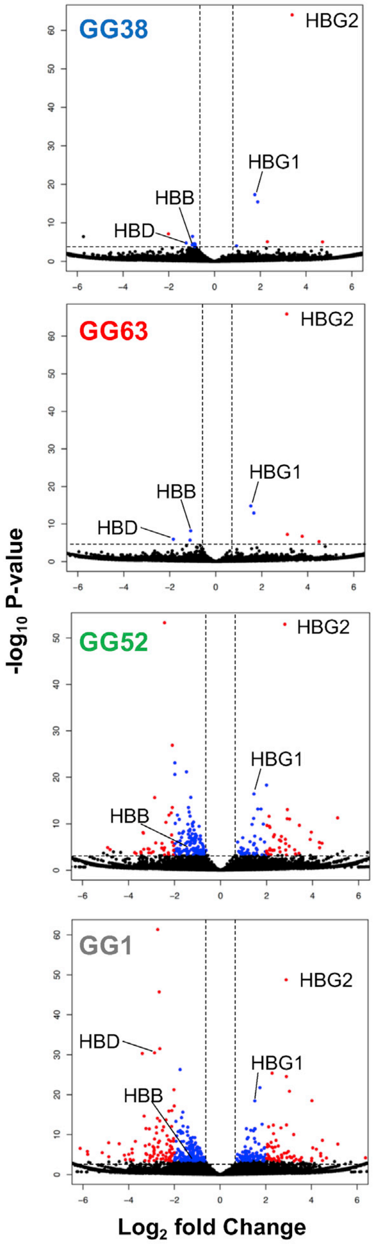

F

Zinc finger construct	Differentially expressed genes (absolute fold change greater than 1.5 and FDR<0.05)
GG38	33
GG63	17
GG52	409
GG1	677

(legend on next page)

using an artificial "looping factor." Specifically, designer zinc-finger (ZF) proteins engineered to bind to the murine β-globin promoter were fused to the dimerization domain (DD) of the GATA1 cofactor Ldb1 (ZF-Ldb1). Ldb1 was chosen because it had been shown to be essential for enhancer-promoter contacts at the β-globin locus.[11] ZF-Ldb1 was capable of promoting LCR-promoter contacts and augmenting transcription.[17] Similar results were obtained when tethering Ldb1 via dCas9.[18] Moreover, ZF-mediated tethering of Ldb1 to the *HBG* genes in adult human erythroid cells increased *HBG* transcription at the expense of *HBB* and *HBD* transcription,[19,20] consistent with a model in which *HBG* and *HBB*/*HBD* genes may compete for LCR activity[21–24] (Figure 1A). Together, these results suggested that engineering genome folding to rewire enhancer-promoter contacts might be a strategy for reactivation of the *HBG* genes to treat SCD.[25]

The aforementioned studies were carried out with cultured erythroid cells *in vitro*. Here we extended these proof-of-concept studies to preclinical murine and non-human primate models with the goal to examine whether the forced reshaping of enhancer-promoter contacts might be a viable strategy to alter gene expression for therapeutic purposes.

RESULTS

Testing of ZF-Ldb1 constructs *in vitro*

ZF-Ldb1 constructs containing the DD of Ldb1 fused to the ZF domain were inserted upstream of an IRES-GFP cassette into a vector containing the promoter of the ankyrin (*ANK1*) gene to drive erythroid-specific expression. We tested four different ZF moieties targeting both *HBG* genes (*HBG1* and *HBG2*, which are adjacent to each other and are thought to have arisen through gene duplication) – GG23 (pcl20-ANK1-GG23DD-IRES-GFP), GG38 (pcl20-ANK1-GG38DD-IRES-GFP), GG52 (pcl20-ANK1-GG52DD-IRES-GFP), and GG63 (pcl20-ANK1-GG63DD-IRES-GFP), with each ZF targeting different areas of the *HBG* promoters (Figure 1B). When tested in fetal-type K562 cells, all ZF-Ldb1 constructs bound to their targets, albeit with varying intensities (Figure 1C).[17,19,20] To test the ability of ZF-Ldb1 to induce HbF synthesis in primary adult erythroid cells, we lentivirally transduced human CD34$^+$ cells and differentiated them toward the erythroid lineage in a three-phase culture system, as described previously.[20,26] Significant induction of γ-globin (the product of the *HBG* genes) in GFP$^+$ cells was achieved with all tested vectors, as measured by cation-exchange high-performance liquid chromatography (HPLC) (Figure 1D). The GG38-, GG63-, and GG52-based constructs were the strongest HbF inducers, with levels similar to those achieved previously with a ZF construct termed GG1-Ldb1, which targets the same region of the *HBG* promoter as does GG63.[19] There was no direct correlation of ZF-Ldb1 binding intensity, as measured by chromatin immunoprecipitation (ChIP) in K562 cells and HbF induction in CD34$^+$ cells, which might be due to different binding site distances from the transcriptional start sites or differences between K562 cells and primary human cells.

To determine which ZF-Ldb1 constructs maximized induction of *HBG* while minimizing off-target effects, RNA sequencing (RNA-seq) was performed on day 13 of *in vitro* erythroid differentiation of human primary CD34$^+$ cells and compared with GFP empty vector-transduced cells. GG38, GG63, GG52, and GG1 increased *HBG1/2* mRNA levels with a concomitant decrease in the mRNA levels of the adult type genes *HBB* in all samples and *HBD* in GG38-, GG63-, and GG1-treated samples (Figure 1E). Importantly, GG38 and GG63 showed the fewest differentially expressed genes (DEG = 33 and 17, respectively; absolute fold change >1.5, false discovery rate [FDR] < 0.05) compared with GG52 (DEG = 409) or GG1 (DEG = 677) (Figure 1F; Tables S1–S4). Thus, we focused the remainder of our studies on the GG38-Ldb1- and GG63-Ldb1-expressing vectors.

Forced LCR-*HBG* chromatin looping increases *HBG* expression in Berkeley (BERK) mice

Our previous work in human cell cultures showed that forced LCR-*HBG* contacts can stimulate *HBG* transcription in a manner well tolerated by the cells.[19,20] To test the efficacy of this approach in a whole-animal model, we employed the BERK mouse model. BERK mice lack murine α- and β-type globin genes and instead carry a transgene that includes a compressed form of the human LCR and the human *HBA*, *HBG*, *HBD*, and *HBB* genes, the latter containing the SCD mutation.[27] The impaired health of homozygous transgene-carrying animals results in poor breeding and intolerance to myeloablative conditioning, which is required for autologous bone marrow transplantation. However, heterozygous animals are healthier, enabling transplantation studies. We tested the efficacy of the GG38- and GG63-carrying vectors by performing myeloablative transplantation of mouse bone marrow lentivirally transduced with either GFP-only control or GG38/GG63-Ldb1-GFP constructs into heterozygous (mouse β^A human β^S) BERK mice, followed by analysis

Figure 1. *In vitro* testing of ZF-Ldb1 constructs

(A) Model of controlling chromatin looping via ZF-Ldb1 constructs to reprogram the β-globin locus (modified from Deng et al.[19]). Red$^+$ indicates the relative degree of gene expression. (B) Specific target sites within the β-globin locus for each of the ZF-Ldb1 constructs. (C) Anti-HA ChIP-qPCR performed in K562 cells shows the specificity of all zinc-finger (ZF) constructs to the *HBG* locus. Data are displayed as signal in the immunoprecipitation (IP) fraction compared with total input; n = 1. (D) Expression levels of HbF as a percentage of total globins (HbF + HbA) by cation-exchange HPLC of GFP$^+$ cells on day 15 of CD34$^+$ *in vitro* culture following lentiviral transduction of CD34$^+$ primary human cells with either GFP control or ZF-Ldb1 constructs. n = 2–3 independent donors. Statistical analyses were done using one-way ANOVA. Error bars represent standard deviation. ns, not significant; *p < 0.05; **p < 0.01. (E) RNA-seq analysis of *in-vitro*-differentiated erythroid cells on day 13 of culture following lentiviral transduction of CD34$^+$ primary human cells with either GFP control or ZF-Ldb1 constructs. n = 2 independent donors. Note that multiple genes may be overlapping in this visualization. Red dot, absolute fold change > 4 and false discovery rate (FDR) < 0.05; blue dot, absolute fold change between 1.5 and 4 and FDR < 0.05; black dot, FDR > 0.05 (not significantly changed). (F) Number of DEGs (absolute fold change greater than 1.5 and FDR < 0.05) among ZF constructs. TSS, transcription start site; HS2, DNAse hypersensitivity site 2 of the LCR; *HBE*, ε-globin; *HBG*, γ-globin; *HBD*, δ-globin; *HBB*, β-globin; LCR, locus control region.

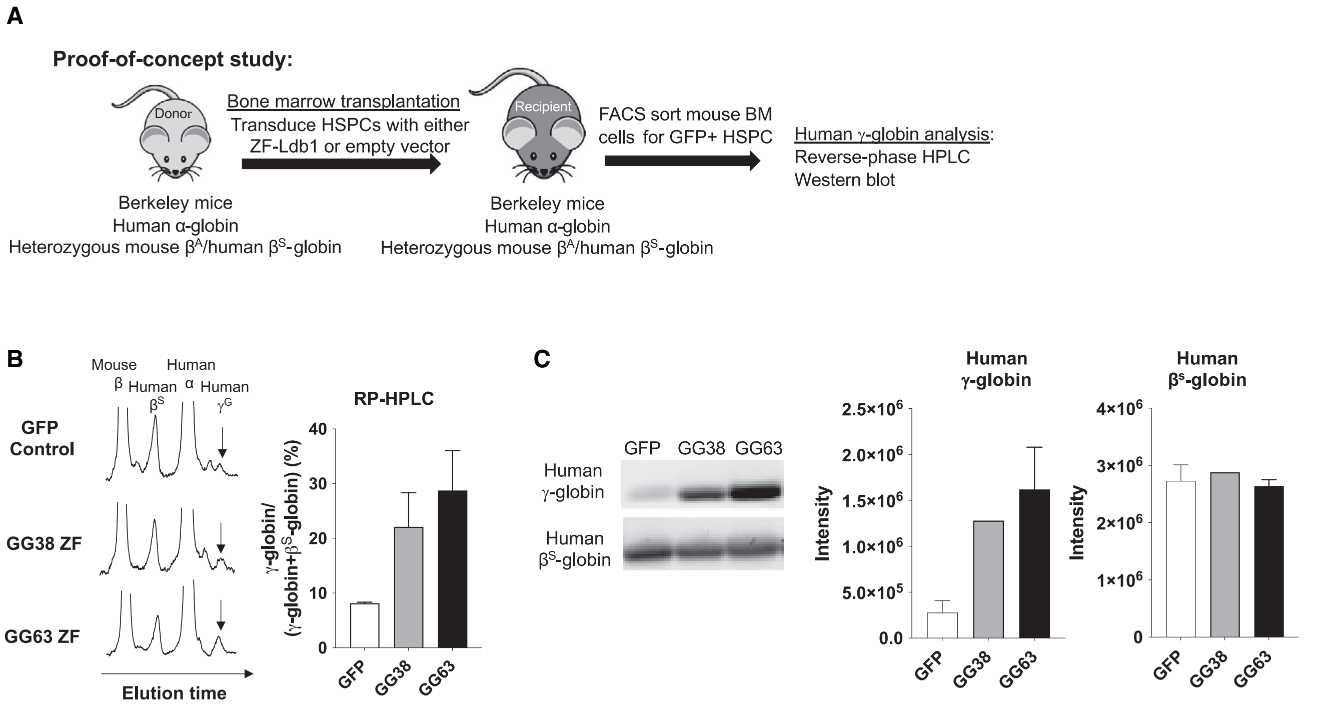

Figure 2. Forced chromatin looping in the humanized BERK mouse model drives *in vivo* induction of γ-globin expression

(A) Experimental design detailing myeloablative transplantation of lentivirally transduced syngeneic mouse bone marrow with either GFP control or GG38/GG63-Ldb1-GFP constructs into heterozygous (mouse β^A human β^S) BERK mice, followed by analysis of HbF and γ-globin expression 10 weeks post transplantation. (B) RP-HPLC analysis and quantification of GFP^+ sorted bone marrow cells from GFP control-, GG38-, or GG63-transplanted mice. n = 2–3 transplanted mice for each condition for RP-HPLC quantification. Error bars represent standard deviation. (C) Representative western blot analysis and quantification of γ-globin in GFP^+ sorted cells from GFP control-, GG38-, or GG63-transplanted mice. n = 1–2 transplanted mice for each condition for western blot quantification. Error bars represent standard deviation.

of HbF and *HBG* expression 10 weeks post transplantation (Figure 2A). Reverse-phase HPLC analysis of GFP^+ bone marrow cells showed a 2.7-fold induction of HbF in GG38-transplanted mice and a 3.6-fold induction in GG63-transplanted mice (Figure 2B). Accordingly, western blot analysis revealed significantly increased expression of γ-globin in the GG38 and GG63 cohorts (Figures 2C and S1). Because the BERK mice used in this study were heterozygous for the human globin genes with minimal symptoms of SCD, it precluded examination of disease parameters, such as erythroid cell indices, spleen size, or urine concentration. Taken together, these data reveal that forced chromatin looping can be employed to activate *HBG* expression in a whole-animal model.

Optimization of ZF-Ldb1 constructs for use in rhesus macaques

Recent advances in lentivirus-based vector development for red cell disorders suggest that addition of the *BCL11A* +58 enhancer enhances erythroid-specific expression of transgenes *in vivo*.[28] We therefore paired the *BCL11A* +58 enhancer with either the *ANK1* or glycophorin A (*GPA*) promoter to drive expression of the GG38/GG63-Ldb1 constructs (Figure 3A). In addition, the vectors contained a P2A sequence followed by a Venus or mEmerald cassette, allowing monitoring of gene expression by flow cytometry. Primary human $CD34^+$ cells from two independent healthy donors were transduced with the vectors at MOIs of 20 or 100, matured *ex vivo* using a two-phase erythroid differentiation culture system, and analyzed for transgene expression by flow cytometry and for HbF induction by HPLC (Figure 3B). Transduced cells displayed no alterations in viability, as determined by flow cytometry (Figure S2). Furthermore, the *ANK1* promoter led to more sustained transgene expression at later stages of erythroid differentiation, regardless of the construct expressed (Figure S2). HPLC analysis on days 9 and 12 showed that the *ANK1* promoter-driven constructs produced the highest levels of HbF for both ZF constructs, with HbF levels of nearly 40% seen with the GG38 construct and 40%–50% with the GG63 vector (Figures 3C and S3). Notably, the +58/ANK1-GG63-P2A-mEmerald vector and the +58/ANK1-GG38-P2A-Venus vector triggered significant HbF induction (33%–66% HbF, 2.5-6-fold HbF induction) with high sustained transgene expression ($Venus^+$ 33%–70%) and vector copy numbers (VCNs) (1.5–4.3) (Figures 3C, S2, and S3), suggesting that these two vectors were the best for further use.

ZF-Ldb1 constructs robustly induce HbF in adult rhesus erythroid cells *ex vivo*

To test whether HbF levels could be raised via forced enhancer-promoter looping in a non-human primate model, we tested our

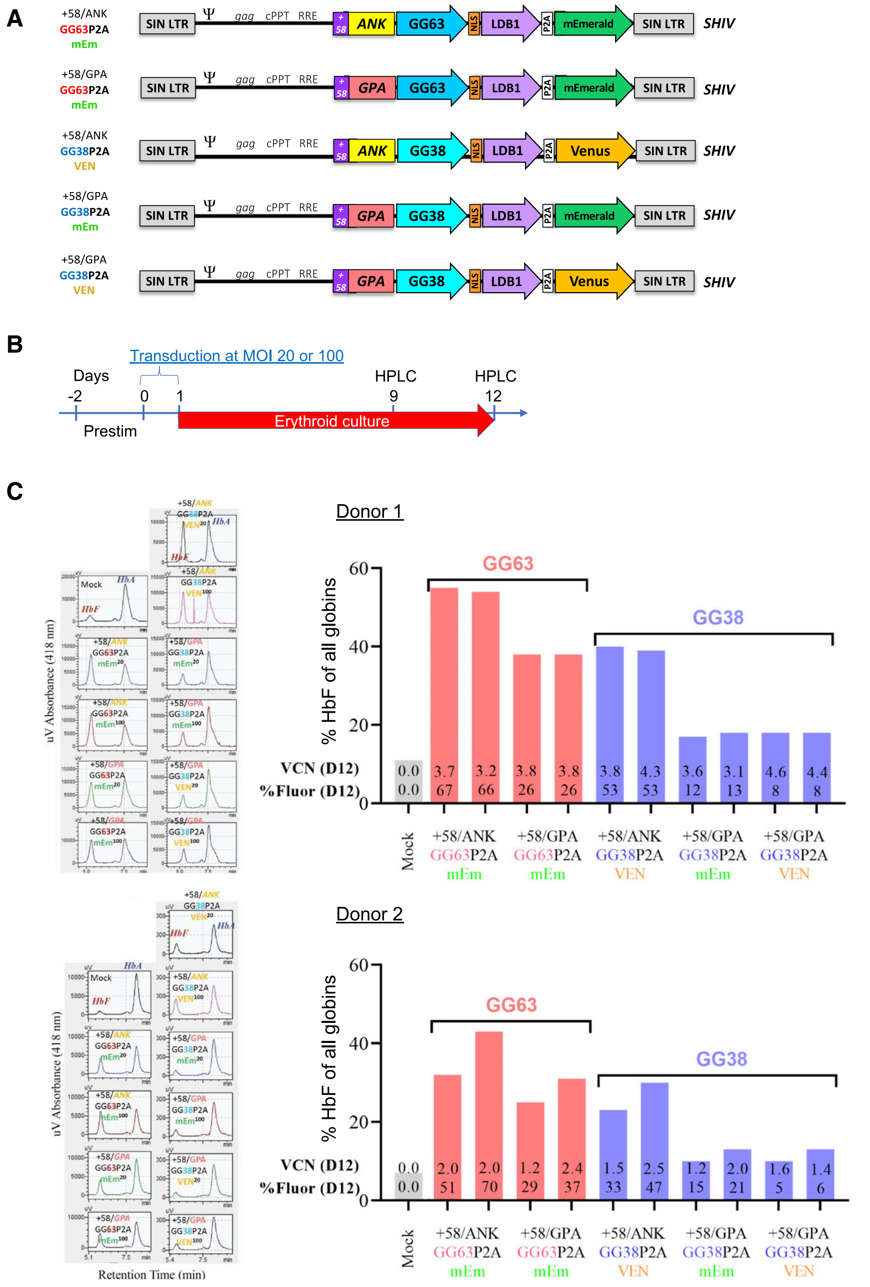

Figure 3. Optimization of ZF-Ldb1 constructs for use in rhesus macaque transplantation

(A) Schematic of ZF-Ldb1 constructs. (B) Experimental design for testing HbF induction of ZF constructs. Adult CD34$^+$ human cells were transduced at an MOI of 20 or 100 on day 0, and HbF was measured by HPLC analysis on days 9 and 12 of *in vitro* erythroid differentiation culture. (C) HPLC analysis following transduction of ZF constructs in two independent CD34$^+$ donor cells on day 12 of culture. HPLC elution tracings (left) and quantification of HPLC peaks (right) illustrate high levels of HbF induction. Vector copy number (VCN D12) and percent Venus/mEmerald positivity (%Fluor D12) are displayed for each vector tested. n = 2 independent donors.

vectors in cultured rhesus macaque cells. Rhesus macaque CD34$^+$ progenitor cells were isolated from granulocyte colony-stimulating factor (G-CSF)-mobilized peripheral blood (PB) as reported previously,[29] transduced with either the +58/ANK1-GG63-P2A-mEmerald or +58/ANK1-GG38-P2A-Venus vector, and differentiated toward erythroid cells. Hemoglobin electrophoresis and reverse-phase (RP)-HPLC showed strong HbF elevation (22.4%–49.1%) compared with non-transduced control (NTC; 6.9%) and control GFP-only-expressing (GFP; 10.9%) cells (Figure 4A). To confirm these results with an alternative source of progenitor cells, we transduced rhesus macaque erythroblasts derived from peripheral blood mononuclear cells (PBMCs) with these vectors, followed by *in vitro* erythroid differentiation. Resulting HbF levels ranged from 48.4%–82.8% compared with GFP only (38.3%) and NTC (32.6%) (Figure S4A). In CD34$^+$ hematopoietic stem and progenitor cells (HSPCs) and PBMC-derived erythroid cells, *ANK1* promoter- and GG38-containing constructs induced higher HbF levels than GPA promoter- and GG63-containing constructs (Figures 4A and S4A). Similarly, the mEmerald or Venus signal was lower in GPA groups compared with *ANK1* groups (Figures 4B and S4B). These results identified the ANK1-GG38 construct as the most robust in terms of expression and HbF induction.

ZF-Ldb1 constructs induce HbF in transplant-derived erythroid cells *in vivo*

Because the ANK1-GG38 combination was found to be superior in terms of *HBG* induction, we used this construct in autologous myeloablative rhesus macaque transplants. After transduction of G-CSF- and plerixafor-mobilized CD34$^+$ HSPCs, a fraction of cells was frozen, and another fraction was differentiated toward the erythroid lineage *in vitro* and examined for VCN and *HBG* expression (Figure S5). While the percentage of Venus$^+$ cells was relatively low (9.96%–14.70%) in differentiated cells, the VCN ranged between 4.3–7.8, and γ-globin levels (45.6%–46.5%) were elevated compared with NTC groups (15.9%–20.9%) (Figures 4C and 4D). Two animals, ZM31 and ZL52, were infused with 3.22–4.72 × 10^6 CD34$^+$ HSPCs/kg and engrafted with typical reconstitution kinetics (Table 1). Blood parameters in transplanted animals were within normal ranges after engraftment throughout the study (Figure S6).

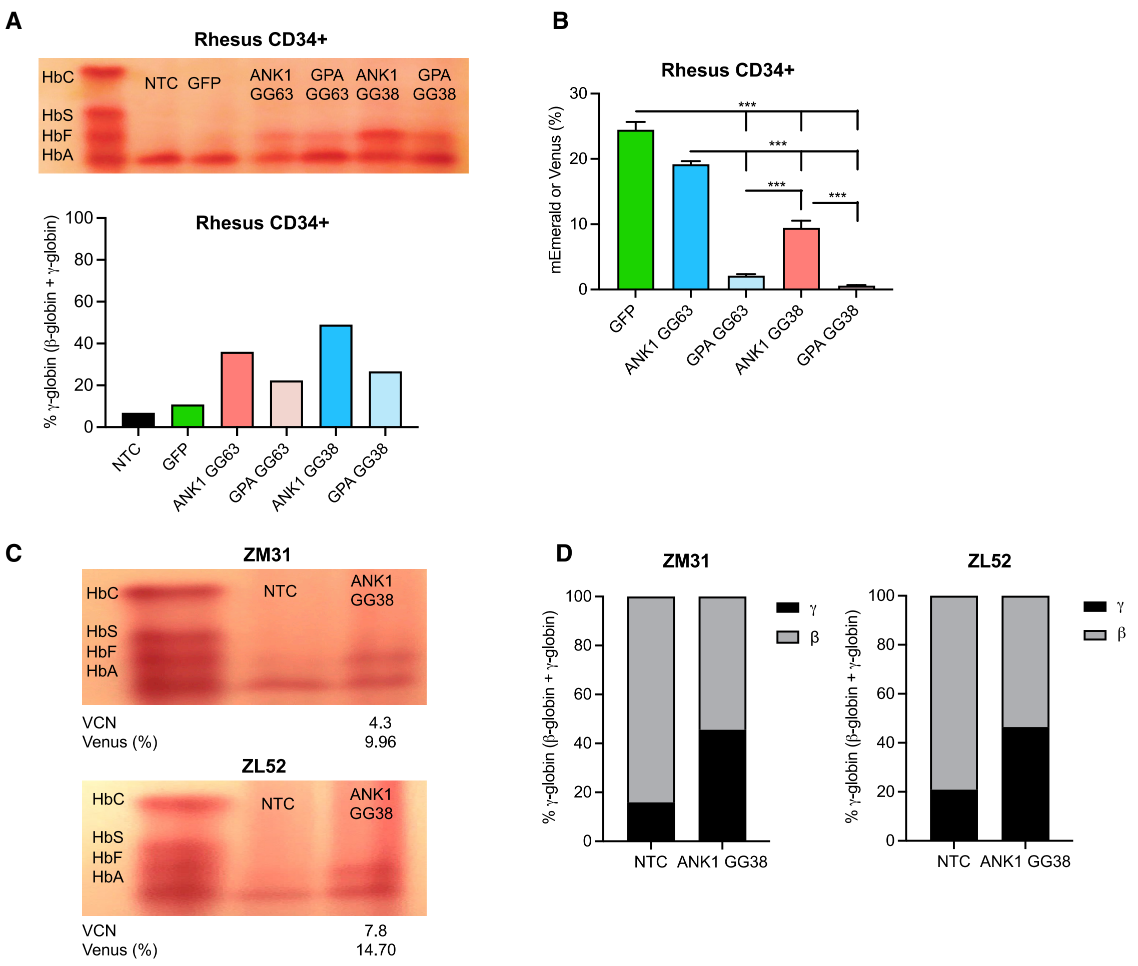

Figure 4. Robust *ex vivo* γ-globin induction in rhesus macaque progenitor cells transduced with optimized ZF constructs

(A) Hemoglobin electrophoresis (top) and RP-HPLC (bottom) in differentiated rhesus macaque CD34$^+$ HSPCs; n = 1. (B) Flow cytometric analysis of mEmerald or Venus vector expression in differentiated rhesus macaque CD34$^+$ HSPCs. Cells were transduced with a lentivirus (MOI = 50 for CD34$^+$ and MOI = 10 for PBMCs) at high cell density (2 × 10^6 cells/mL) in XVIVO-10 medium + SFT (100 ng/mL each); n = 3. Statistical analyses were done using one-way ANOVA. Error bars represent standard deviation. ***$p < 0.001$. (C and D) *Ex vivo* analysis of rhesus macaques receiving transplants, showing (C) hemoglobin electrophoresis and (D) RP-HPLC analysis of non-transduced cells (NTCs) and GG38-expressing lentivirus-transduced cells on day 14 of erythroid differentiation of infused products; n = 1. HbF and flow cytometry assays were performed on unsorted bulk erythroid cells. HbC, hemoglobin C; HbS, hemoglobin S/sickle hemoglobin; HbF, hemoglobin F/fetal hemoglobin; HbA, hemoglobin A/adult hemoglobin.

Long-term increases in HbF levels in ZF-Ldb1-expressing erythroid cells

After cell infusions, PB samples were collected regularly to evaluate VCNs in cell fractions, HbF$^+$ cell (F-cell) percentage, and γ-globin expression in erythroid cells from transplanted animals. Stable VCNs in myeloid (~0.3 for ZM31, ~0.6 for ZL52) and lymphoid (~0.3 for ZM31, ~0.8 for ZL52) cells were detected at 135 weeks after transplantation (Figure 5A). In bulk analyses, F-cells (Figure 5C) and γ-globin expression (Figure 5D) were high only early post transplantation but receded to control animals' levels 30–45 weeks post transplantation, consistent with decreased Venus reporter expression (Figure 5B). Although the VCNs were considerable, Venus$^+$ erythroid cells were limited (1%–3%) and not substantially different than in other cell fractions, including myeloid and lymphoid cells for both animals (Figure 5B), suggesting that decreased transgene expression was not simply a consequence of loss of Venus expression in enucleated circulating erythroid cells. To investigate whether ZF-Ldb1 expression was toxic to erythroid cells, we evaluated the VCN in CD20$^+$ B-cells, CD45$^-$CD71$^+$ erythroid progenitors, and all other CD45$^+$ cells (CD45$^+$CD20$^-$) from bone marrow mononuclear cells of the transplanted animals. The results show that erythroid progenitors had VCNs comparable with those in other cell lineages tested, suggesting that ZF-Ldb1 expression was not selectively toxic to erythroid progenitors (Figure S7).

Although ZF-Ldb1 was not stably expressed over time in bulk populations, we hypothesized that the subset of cells expressing ZF-Ldb1 (Venus$^+$ cells) might still respond to its activity. We sorted Venus$^+$

Table 1. Summary of the transplanted rhesus macaques

Animal	ZM31	ZM52
Age at transplantation, years/sex	4.2/female	5.2/female
Infusion product, cells/kg	3.22×10^6	4.72×10^6
Vector	ANK1-GG38	ANK1-GG38
Transduction MOI/cell concentration	$50/2 \times 10^6$/mL	$50/2 \times 10^6$/mL
Infusion product VCN	4.3	7.8
Neutrophil engraftment day (1,000/mL)	11	10

erythroid cells from PB of the transplanted animals and measured *HBG* expression approximately 1 year post transplantation. Reporter-positive red blood cells (RBCs) expressed significantly higher *HBG* levels compared with Venus$^-$ cells for the respective animal 1 and 2 years post transplantation (5.9%–22.8% in Venus$^+$ RBCs versus 0.3%–1.2% in Venus$^-$ RBCs; Figure 6A). We investigated whether there was any difference in γ-globin expression relative to the degree of Venus expression. We sorted Venus-mid and Venus-high populations approximately 2 years post transplantation and evaluated γ-globin expression levels by RP-HPLC. No differences were observed between mid- and high-Venus-expressing populations (Figure 6B). Nevertheless, these data indicate the long-term functionality of the ZF-Ldb1 construct to induce HbF expression and are consistent with sustained HbF induction in GFP$^+$ cells in our BERK murine transplant studies utilizing the GG38-Ldb1 and GG63-Ldb1 constructs (Figure 2). Taken together, our data suggest that forced enhancer-promoter rewiring in murine and macaque models allows significant increases in *HBG* gene expression.

DISCUSSION

Our prior work showed that ZF-mediated tethering of Ldb1 to the *HBG* promoter can reactivate its expression in normal and SCD patient-derived cultured adult erythroblasts.[19,20] More recently, a study using transcriptional activator-like effectors (TALEs) to tether Ldb1 to the *HBG* promoters has shown similar results.[30] Here, we find that the optimization of our ZF-Ldb1 vector achieved HbF induction comparable with our prior studies[19,20] (Figure 1D) while reducing off-target effects on the transcriptome (Figures 1E and 1F). Addition of the *BCL11A* +58 enhancer in combination with the *ANK1* promoter and the GG38 and GG63 ZF moieties achieved considerable HbF expression in rhesus macaques and human primary cells. Furthermore, we show for the first time in two independent animal models that forced proximity of the LCR with the *HBG* promoters augments HbF expression *in vivo*. A naturally occurring gain-of-function mutation at the *HBG* genes that triggers recruitment of the Ldb1 complex causes elevated *HBG* expression throughout adult life,[31] providing genetic evidence that Ldb1 tethering to the *HBG* genes may be a potent means to reactivate them.

Although the number of F-cells and HbF levels were significantly upregulated in the first 3 months post transplantation in rhesus macaques, levels gradually decreased and stabilized to around pre-transplantation levels 30–40 weeks after transplantation. VCNs in the transplanted animals remained stable throughout the 135 weeks of follow up. Thus, the drop in F-cells and HbF levels was not due to loss of vector-containing cells over time. Although it is established that transplantation itself induces a transient rise in HbF in early transplantation periods,[32] we have demonstrated previously that transplantation of rhesus macaques with CD34$^+$ HSPCs transduced with a GFP vector induced lower levels (2.53%) of HbF only during the early phase of transplantation,[33] suggesting that ZF-Ldb1 expression contributes to the observed HbF induction. This notion is supported by Venus$^+$ RBCs having 8%–24% *HBG* levels, while Venus$^-$ RBCs displayed *HBG* expression levels similar to controls.

One major limitation of the study is that Venus expression remained low despite high VCNs (Figure 5). This is potentially due to low gene expression in rhesus cells from use of a polycistronic vector with Venus in the third position[28] or gene silencing, possibly via DNA methylation of the integrated vector. Decreased expression *in vivo*, perhaps because of positional effects,[34] could explain why some cells continue to have Venus expression long term, while others do not express detectable Venus reporter despite VCN data suggesting that most of the cells carry the vector. We have shown recently that while shRNA-mediated *BCL11A* knockdown in rhesus macaques induced HbF only transiently, sustained shRNA expression and increased HbF production was achieved by incorporating a truncated form of the human erythropoietin receptor (thEpoR) into the vector.[28] thEpoR expression provides a selective advantage to transduced erythroid precursor cells. We envision that this approach might be similarly beneficial in our system to achieve sustained ZF-Ldb1 expression *in vivo*.

The use of clinically relevant vectors,[35] the identification of key regulatory regions, including the *BCL11A* +58 erythroid-specific enhancer,[36,37] and the application of the *ANK1* insulator-promoter in vector development[38] have driven much of the initial optimization shown in this study. Further improvement of the forced chromatin looping strategy to achieve robust, sustained, and pancellular expression might provide an avenue for future therapeutic applications. Tethered Ldb1 is capable in non-erythroid cells, such as neuronal progenitor cells, of efficiently generating long-range contacts between genomic segments much farther apart than at the β-globin locus (∼500 kb),[39] suggesting that forcing long-range chromatin contacts via Ldb1 tethering might be more broadly applicable.

Taken together, our findings serve as a proof of concept demonstrating that forced chromatin looping may be developed to become a useful tool for treatment of genetic disorders. ZF domains may be less antigenic compared with TALEs or Cas9, can be engineered to a high level of target specificity, and can be used to tether protein moieties that, in turn, reshape chromosome conformation. In this manner, via engagement of a powerful distal enhancer, such as the LCR, gene expression changes may be increased to levels not achievable by simple fusion to ZF moieties of conventional transactivation domains. We envision that altering nuclear architecture in a manner that re-connects enhancers with their natural target genes might be applicable to diseases in which enhancer-promoter

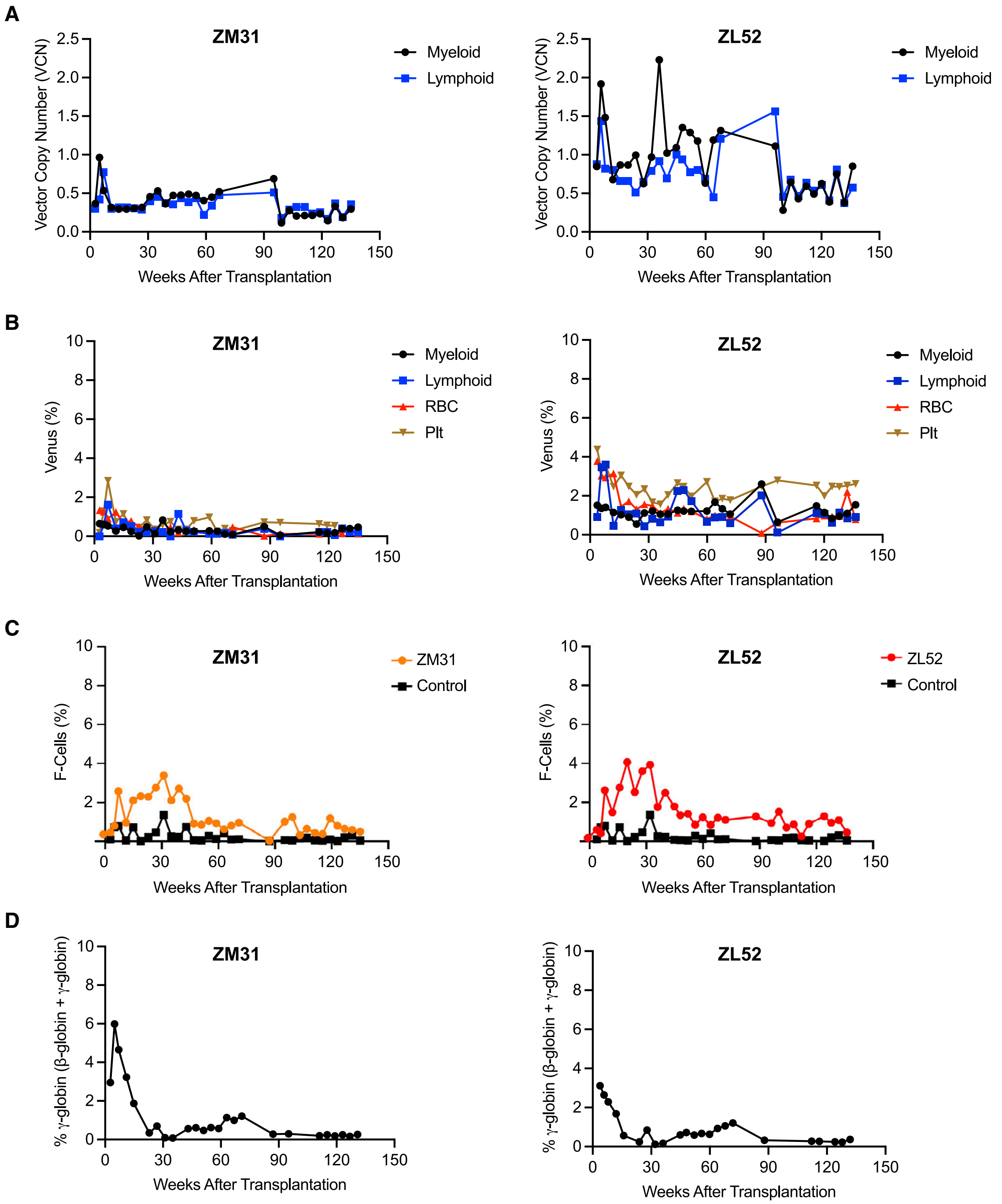

Figure 5. Long-term stable HbF expression was not seen in analysis of bulk peripheral blood from transplanted rhesus macaques
(A–D) Analysis of PB from rhesus macaques (n = 2) transplanted with the optimized ANK1-GG38 ZF construct: (A) VCN, (B) Venus (percent), (C) F-cells (percent), and (D) γ-globin (percent) in transplanted animals. n = 2 independent transplanted rhesus macaques. Control: red blood cells (RBCs) from non-transplanted animals.

connectivity is disrupted. This includes, for example, developmental disorders in which chromosomal abnormalities affect chromatin domain boundaries, resulting in inappropriate enhancer-promoter contacts.[40]

Recent preclinical and patient-based studies have shown that gene therapy may be feasible in the fetal stages of life, as being developed in the field of neurodegenerative disease,[41] as well as in the adult stages, as most notably embodied by use of gene addition therapy

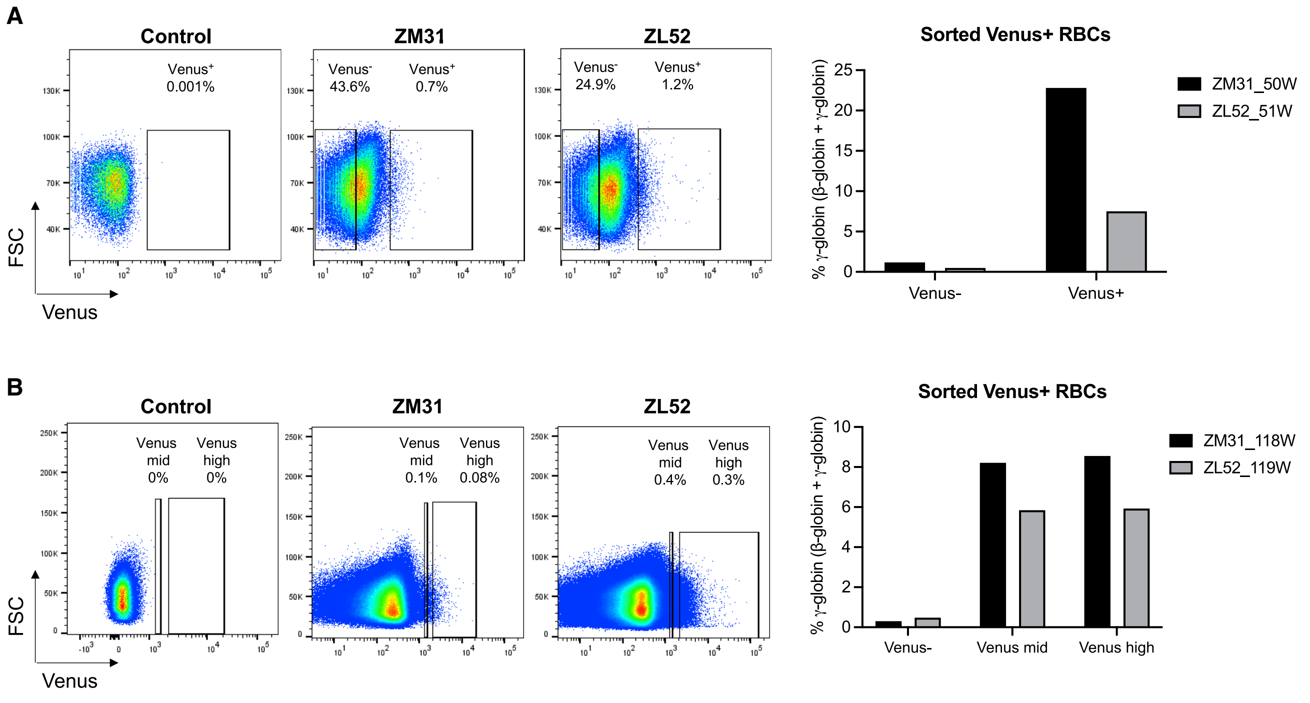

Figure 6. Significant *in vivo* γ-globin induction in Venus+ RBCs from transplanted rhesus macaques

(A and B) Sorted Venus+ red blood cells (RBCs) displayed high γ-globin protein expression compared with Venus− RBCs. Shown are the percentage of γ-globin expression (expressed as γ-globin / [β-globin + γ-globin]) in sorted RBCs at (A) 1 year post transplantation and (B) 2 years post transplantation. n = 2 independent transplanted rhesus macaques.

for the treatment of β-thalassemia major[42,43] and SCD.[44] Recent studies have identified potential therapeutic targets for rewiring of enhancer-promoter interactions, including *EPHA4*-related limb malformations[40] and *SOX9*-mediated craniofacial disorders,[45] among others.[46] One advantage of forced enhancer-promoter rewiring might be that it not only reduces expression of the mistargeted gene but also restores expression of the natural enhancer target gene. Thus, advances such as those described here may help to allow targeted treatment of enhanceropathies across the age spectrum for a wide variety of inherited and acquired disorders that involve mis-wired or dysfunctional enhancers.

It is important to note that advances in gene editing/manipulation technologies have yielded attractive new approaches for the treatment of SCD, including altering the expression of *BCL11A*[47,48] or the *HBG* genes directly,[24,49,50] or to convert the SCD mutation into a harmless variant.[51] In light of these developments, we are not proposing to use forced chromatin looping as a therapeutic modality in this context. Instead, we provided proof of concept that rewiring of enhancer-promoter contacts might be a means to alter gene expression in settings where regulatory elements or genome architectural features are disrupted. That said, there are additional considerations that might make forced chromatin looping an advantageous strategy. Redirecting an enhancer away from its original target is expected to dampen transcription of the target gene. In the case of the β-globin cluster, our prior work has demonstrated that the gain in *HBG* transcription was accompanied by a reciprocal reduction in *HBB* expression.[19] In the case of SCD, in which a mutant form of *HBB* is the root cause of the disease, this approach combines the benefits of increased HbF levels with a reduction of the toxic form of *HBB*. These combinatorial effects might be beneficial when considering this strategy for the treatment of inherited diseases and enhanceropathies in cases where gene editing is not applicable.

MATERIALS AND METHODS

Animals

Mice were maintained at the Children's Hospital of Philadelphia (CHOP) animal facility, and all experiments were carried out using protocols approved by the CHOP Institutional Animal Care and Use Committee. BERK sickle cell mice carrying the sickle transgene (*Hba*^{tm1Paz} *Hbb*^{tm1Tow} Tg(HBA-HBBs)41Paz/J) were obtained from the Jackson Laboratory (003342).

Rhesus macaques (*Macaca mulatta*) were used following the guidelines set out by the Public Health Service Policy on Humane Care and Use of Laboratory Animals under a protocol (H-0136) approved by the Animal Care and Use Committee of the National Heart, Lung, and Blood Institute (NHLBI).

Cells, cell culture, lentiviral transduction, and cation-exchange HPLC

K562 cells were grown in RPMI medium supplemented with 10% fetal bovine serum (FBS) and 1% penicillin-streptomycin. PBMCs

were obtained from the University of Pennsylvania Human Immunology Core (Philadelphia, PA, USA). $CD34^+$ cells were purified by using a MACS MicroBead Kit (Miltenyi) and cultured in a 3-phase *in vitro* culture, as described previously.[52] Lentiviral stocks were generated by co-transfection of the previously described gene transfer plasmids (pcl20-ANK1-GFP, pcl20-ANK1-GG23DD-IRES-GFP, pcl20-ANK1-GG38DD-IRES-GFP, pcl20-ANK1-GG63DD-IRES-GFP, pcl20-ANK1-GG52DD-IRES-GFP, or pcl20-ANK1-GG1DD-IRES-GFP[19]) together with the envelope plasmid (vesicular stomatitis virus [VSV]-G), the packaging plasmid (pMDLg/p RRE), and the pRSV-REV plasmid into HEK293T cells, and the viral supernatant harvested and concentrated by ultracentrifugation using a protocol described previously.[20] The ZF proteins tested in the present study bind to sequences located at −443 bp (GG23), −182 bp (GG38), −114 bp (GG63 and GG1), and −45 bp (GG52) with respect to the *HBG1/2* transcription start sites, following previously established design principles,[19,53] including target sites located in accessible chromatin as well as at sites where there are no known transcription factor binding sites. Specifically, the tested ZF constructs target the following sequences located in the γ-globin promoter region: GG23, 5′-TTAGGCATAG GTCCAGGATT-3′; GG38, 5′-TTGAGATAGTGTGGGGAAGG-3′; GG63 and GG1, 5′-GGTCAAGGCAAGGCTGGCCA-3′; GG52, 5′-A GCCGCCGGCCCCTGGCCTC-3′. Viral titration was performed using K562 cells and titered to the virus transduction unit of 1E8–1E9 particles/mL. K562 or CD34 cells were spin-infected at 2,250 rpm at room temperature for 1.5 h as described previously,[19] and GFP^+ cells were sorted by fluorescence-activated cell sorting (FACS) on day 2 post infection. Cation-exchange HPLC for quantification of HbF was performed as described previously.[52,54]

ChIP

Anti-hemagglutinin (HA) ChIP-qPCR was performed in K562 cells using human-specific primers for HS2, *HBE*, *HBG*, *HBD*, *HBB*, and *CD4* as described previously.[19]

RNA extraction, qRT-PCR, and RNA-seq

RNA samples were harvested in TRIzol (Thermo Fisher Scientific) and purified with the RNeasy Mini Kit (QIAGEN). For RNA-seq, 200 ng RNA, isolated as just described, was depleted for ribosomal RNA by using the Ribo-Zero removal reagents and protocol from the ScriptSeq Complete Kit (Illumina), followed by purification with the RNeasy MinElute Clean Up Kit (QIAGEN). Sequencing libraries were constructed and analyzed, libraries were sequenced in paired-end mode, and reads were processed as described previously.[26,55]

Murine bone marrow isolation and $lineage^-$ $Sca1^+$ $ckit^+$ (LSK) sorting

All BERK mice used for experiments were heterozygous for the sickle transgene (mouse β^A human β^S), confirmed by RP-HPLC with appropriate mouse and human globin controls. Male BERK mice were euthanized at 2–3 months of age by CO_2 narcosis. Cells from bone marrow were harvested by flushing the tibiae, femora, and hip bones with PBS containing 0.5% BSA and 2 mM EDTA. $Lineage^-$ cells from total bone marrow were isolated using the Lineage Cell Depletion Kit (Miltenyi Biotech, catalog number 130-090-858) according to the manufacturer's protocol. $Lineage^-$ cells were stained with APC-cKit (Thermo Fisher Scientific, catalog number 17-1171-83) and PE-Sca1 (BD Biosciences, catalog number 553336). LSK cells were sorted on an MoFlo Astrios Sorter (Beckman Coulter, Brea, CA, USA) and then cultured in SFEM medium (STEMCELL Technologies, catalog number 09600) containing 10% FBS (SAFC Biosciences) with 50 μM β-mercaptoethanol (Sigma-Aldrich, catalog number M3148), 20 ng/mL Flt3L, 20 ng/mL interleukin-6 (IL-6), 100 ng/mL stem cell factor (SCF), and 20 ng/mL thrombopoietin (TPO; PeproTech) for 24 h.

Lentiviral transduction and syngeneic bone marrow transplant of heterozygous BERK SCD mice

Cultured LSK cells were transduced with lentiviruses on day 2 using a RetroNectin (T100B, Takara)-based method.[56] Briefly, lentivirus carrying pcl20-ANK1-GFP, pcl20-ANK1-GG38DD-IRES-GFP, or pcl20-ANK1-GG63DD-IRES-GFP were loaded into RetroNectin coated 12-well plates to aim for a transduction efficiency of 25%–30%. Cultured LSK cells were transferred to the lentivirus-loaded plates and incubated for 1 more day. On day 3, 285K LSK cells were mixed with 500,000 Sca1-depleted competitor BM cells and injected retro-orbitally into lethally irradiated (12 Gy, split dose, Orthovoltage Precision X-ray) recipient heterozygous BERK mice. GFP^+ BM cells (constituting 5%–10% of transplanted mouse bone marrow) were isolated via FACSAria-based cell sorting.

RP-HPLC analysis of transplanted BERK mice

For BERK murine transplant analysis, RP-HPLC analyses were conducted as reported previously, with slight modifications.[20,57] Briefly, murine whole bone marrow or GFP^+ bone marrow samples were lysed in 100 μL MilliQ water and centrifuged at 10,000 × *g* for 10 min. A 50-μL aliquot of the supernatant was injected on a Hitachi D-7000 HSM series apparatus (Hitachi Instruments, San Jose, CA, USA) using an Aeris 3.6-μm, Widepore C4 200-Å, liquid chromatography (LC) 100 × 4.6-mm column (Phenomenex, Torrence, CA, USA) and a gradient from 20%–60% acetonitrile in 0.1% trifluoroacetic acid over 25 min with UV detection at 215 nm. Types and relative quantities of hemoglobins in samples were assessed by comparison with standard hemoglobin controls.

Western blot analysis

Western blot analysis was performed as described previously.[26] Briefly, fluorescent western blotting was performed on Immobilon-FL polyvinylidene difluoride membranes (Millipore), blocking was performed in Odyssey blocking buffer (LI-COR Biosciences), and antibody staining was performed in blocking buffer diluted 1:1 in Tris-buffered saline-0.1% Tween 20. Primary staining was performed overnight with gentle shaking at 4°C, and secondary staining was performed for 1 h at room temperature. Primary antibodies included γ-globin (1:1,000 dilution, Novus Biologicals, catalog number NB-110-41084) and β-globin (1:1,000 dilution, Santa Cruz Biotechnology, sc-21757), and secondary antibodies included IRDye 800 donkey anti-goat immunoglobulin G (IgG) (1:15,000 dilution,

LI-COR Biosciences, catalog number 925-32214) and IRDye 680 donkey anti-mouse IgG (1:15,000 dilution, LI-COR Biosciences, catalog number 926-68072). Blots were visualized at 700 and 800 nm on the Odyssey imaging system (LI-COR Biosciences) and quantitated in Image Studio Lite (LI-COR Biosciences).

Vector packaging and lentiviral production

Previously published protocols were modified for use in erythroid ZF vector optimization.[58,59] Briefly, vectors were packaged with chimeric simian human immunodeficiency virus (SHIV), produced in HEK293T suspension cultures, Mustang Q purified (XT5 ion-exchange capsule, Pall Life Sciences), and concentrated by diafiltration in X-VIVO 10 medium (Lonza, Walkersville, MD, USA) to achieve a final concentration of about 50-fold from the starting material. The vectors were then aliquoted and stored at −80°C before titration using HOS cells as described previously.[59]

Vector optimization, transduction, and CD34$^+$ erythroid differentiation testing

Purified CD34$^+$ cells were isolated from G-CSF-mobilized PB of healthy volunteers from the St. Jude Human Applications Laboratory for Key Biologics (Memphis, TN, USA). CD34$^+$ cells were cultured for 2 days (pre-stimulation) in X-VIVO 10 (Lonza) with 100 ng/mL of SCF, Fms-related tyrosine kinase 3 ligand (FLT3-ligand), TPO (CellGenix, Freiburg, Germany), 50 U/mL of penicillin/streptomycin (Corning), 2 mM L-alanyl-L-glutamine (Corning), and 1% recombinant human serum albumin (Grifols Biologics). Cells were transduced with erythroid ZF constructs targeting an MOI of either 20 or 100 in the presence of 8 μg/mL protamine sulfate (St. Jude Children's Research Hospital pharmacy) and 1% recombinant human serum albumin (Grifols Biologics). CD34$^+$ erythroid differentiation of transduced cells was performed using a two-phase system as described previously.[59] Flow cytometry of rhesus macaque mEmerald (with a 515/20 filter, Chroma Technology) and Venus (with a 525/LP filter, BD Biosciences) was performed using a CytoFlex (Beckman Coulter) instrument on days 5, 7, 9, and 12 of differentiation. FlowJo v.10 (FlowJo, Ashland, OR, USA) was used for data analysis and graphic representation. Samples from days 9 and 12 of erythroid differentiation were prepared for cation-exchange HPLC analysis by isolating 100,000 erythroid cells, washing with PBS, lysing in hemolysate reagent (Helena Laboratories, 5125), and centrifuging at 14,000 × *g* for 10 min. Quantification of hemoglobin tetramers and individual globin chains was performed using ion-exchange columns on a Prominence HPLC system (Shimadzu). Proteins eluted from the column were identified at 220 and 415 nm with a diode array detector. The relative amounts of hemoglobins or individual globin chains were calculated from the area under the 415-nm peak, with percentage of HbF = [HbF/(HbA + HbF)] × 100. Types and relative quantities of hemoglobins in samples were assessed by comparison with standard hemoglobin controls.

Rhesus macaque PBMC and CD34$^+$ isolation and transplantation

Rhesus macaque PBMCs were isolated from PB using Ficoll-Paque PLUS density gradient medium (GE Healthcare) following manufacturer recommendations. G-CSF-mobilized (Amgen) and plerixafor-mobilized (Amgen) rhesus macaque CD34$^+$ HSPCs were collected as described previously.[29,60] In short, the animals were treated with a 5-day course of 15 μg/kg G-CSF (Amgen) subcutaneously and a single subcutaneous dose of 1 mg/kg AMD3100 (Sigma-Aldrich) on the morning of the fifth day, 3–4 h before leukapheresis. A small-volume leukapheresis procedure was performed using a CS3000 cell separator (Baxter Fenwal), and CD34$^+$ HSPCs were immunoselected using a rhesus macaque CD34$^+$ antibody (clone 12.8, Fred Hutchinson Cancer Research Center) and an anti-mouse IgM bead (Miltenyi Biotech). A CD34$^+$ antibody (550761, BD Biosciences) was used to determine the purity of the immunoselected population.

Rhesus macaque CD34$^+$ lentiviral transduction, *ex vivo* erythroid differentiation, and transplantation

Rhesus macaque PBMCs and mobilized CD34$^+$ HSPCs were transduced with lentiviral particles expressing ZF constructs using a high-density transduction protocol as described previously.[35] Briefly, progenitor cells were pre-stimulated in serum-free X-VIVO10 medium (Lonza) containing SCF, Flt-3, and TPO (100 ng/mL each, R&D Systems) at a density of 2×10^6 cells/mL for 24 h. Then, the medium was replaced with fresh pre-stimulation medium with growth factors and ZF constructs at MOI 10 and 50 for PBMCs and CD34$^+$ HSPCs, respectively. After 24 h of transduction, the majority of cells were cryopreserved for later transplantation; a small portion of the transduced CD34$^+$ HSPCs were used for RBC differentiation to confirm VCN, reporter gene (Venus), and HbF induction using a previously reported protocol.[61] Briefly, 1×10^5 cells/mL were inoculated into tissue culture plates (Corning) coated with irradiated OP-9 cells (ATCC, Crl-2749) in erythroid proliferation medium consisting of Iscove's modified Dulbecco's medium (Mediatech) with 10 ng/mL SCF, 1 ng/mL IL-3 (R&D Systems), 2 U/mL erythropoietin (EPO, Amgen), 10^{-6} M dexamethasone (VETone), 10^{-6} M estradiol (Pfizer), and 20% FBS (Gibco) for 6 days. Then, cells were incubated for an additional 8 days in maturation medium containing 10 ng/mL insulin (Lilly), 500 mg/mL holo-transferrin (Sigma-Aldrich), 2% (w/v) bovine serum albumin (Roche), 2 U/mL EPO, and 20% FBS. DNA and protein samples were isolated from differentiated cells to determine VCN and γ-globin protein expression, respectively. Lentivirally transduced CD34$^+$ HSPCs (specific cell numbers are listed in Table 1) were then reinfused into rhesus macaques following 5 Gy × 2 total body irradiation at a dose of 0.6 Gy/min using a Co-60 irradiator located at the Armed Forces Radiobiology Research Institute (Bethesda, MD, USA).

Quantitative PCR

Genomic DNA was extracted from *ex-vivo*-differentiated cells, PB myeloid cells, or lymphoid cells isolated using the DNeasy Blood and Tissue kit (QIAGEN) following the manufacturer's protocol. VCNs were measured by a QuantStudio 6 Flex real-time PCR system (Thermo Fisher Scientific) using a self-inactivating long terminal repeat (SIN-LTR) probe and primers and ribosomal RNA probe and primers (TaqMan ribosomal RNA control reagents, Applied Biosystems).[62]

Flow cytometry

For rhesus macaque analysis, rhesus macaque mEmerald and Venus expression in *ex-vivo*-transduced cells or PB cell fractions were analyzed using flow cytometry (FACSCanto, BD Biosciences). The F-cell percentage in PB RBCs of the transplanted animals was determined by a previously published protocol using a primary HbF-specific antibody (551796, BD Biosciences) and an allophycocyanin (APC)-conjugated secondary antibody (550874, BD Biosciences).[33]

Hemoglobin electrophoresis

Hemoglobin content in differentiated PBMCs and CD34$^+$ HSPCs was determined using cellulose acetate membranes according to the manufacturer's instructions (Helena Laboratories, TX, USA).

RP-HPLC analysis of transplanted rhesus macaques

Rhesus PB RBCs or *ex-vivo*-differentiated progenitor cells were washed with phosphate-buffered saline (Corning Cellgro) three times. After lysing RBCs in 25–100 μL HPLC-grade water, cells were centrifuged at 16,000 × g for 10 min. The supernatant was added to 2.5–10 μL of 100 mmol/L TCEP (tris(2-carboxyethyl)phosphine; Thermo Fisher Scientific) and incubated for 5 min at room temperature. Then, the mixture was added to 22.5–85 μL of 0.1% trifluoroacetic acid/32% acetonitrile, and the solution was briefly pulse vortexed three times. The reduced solution (10–40 μL) was analyzed at a 0.7 mL/min flow rate for 58 min using the Agilent 1100 HPLC (Agilent Technologies) equipped with an RP column (Aeris 3.6-μm Widepore C4 200-Å, 250 × 4.6 mm, Phenomenex), and guard cartridge (AJ0-4330, Phenomenex). Solvent A (0.12% trifluoroacetic acid (TFA) in water) and solvent B (0.08% TFA in acetonitrile) were used with a gradient for separation of globin protein of 35% solvent B, followed by changes in the percentage of solvent B as follows: 3 min at up to 41.2%, 3 min at up to 41.6%, 5 min at up to 42%, 4 min at up to 42.4%, 6 min at up to 42.8%, 6 min at up to 44.4%, 6 min at up to 47%, 14 min at up to 100%, and re-equilibration for 11 min at 35%. The globin-chain peaks were detected at 215 nm and confirmed by an Agilent HPLC-6224 mass spectrometer equipped with an electrospray ionization (ESI) interface and a time-of-flight mass detector (Agilent Technologies) as described previously.[38]

DATA AVAILABILITY

RNA-seq data were deposited in the Gene Expression Omnibus with accession number GSE221182. For additional original data requests, please contact the corresponding author.

SUPPLEMENTAL INFORMATION

Supplemental information can be found online at https://doi.org/10.1016/j.omtn.2023.01.016.

ACKNOWLEDGMENTS

This work was supported by National Institutes of Health, National Heart, Lung, and Blood Institute (NHLBI) grant R01HL119479 (to G.A.B.) and National Institute of Diabetes and Digestive and Kidney Diseases (NIDDK) grant K08DK129716 (to S.A.P); Doris Duke Charitable Foundation Physician Scientist Fellowship grant 2020062 (to S.A.P.); an American Society of Hematology Scholar Award (to S.A.P.); the St Jude Children's Research Hospital Collaborative Research Consortium on Novel Gene Therapies for Sickle Cell Disease; research funding from Bioverativ (to G.A.B.) and Pfizer, Inc. (to G.A.B.); and a generous gift from the DiGaetano family. We would like to thank Andreas Reik and Philip D. Gregory (Sangamo Therapeutics) for providing the zinc-finger constructs.[19] We would also like to acknowledge Ross C. Hardison, Cheryl A. Keller, and Belinda Giardine for assistance with RNA-seq analysis; Jeremy D. Grevet and Kiwon Lee for assistance with cell sorting, and Jennifer Yano for assistance with BERK mouse husbandry. This work was also supported by the NHLBI Intramural program. We would like to thank NHLBI and Division of Veterinary Resources (DVR) staff for animal care. The authors gratefully acknowledge research support from the Armed Forces Radiobiology Research Institute.

AUTHOR CONTRIBUTIONS

S.A.P., S.D., J.F.T., and G.A.B. designed the experiments, analyzed the data, and wrote the manuscript with input and editing from M.W., B.R., W.T., and M.J.W. A.C.B., A.E.K., N.S.L., and R.E.D. performed rhesus macaque care, transplantation, and sample derivation. Experiments were conducted by S.A.P., S.D., V.C., B.R., S.K.B., J.J., J.W.R., R.E.T., N.U., A.L., K.E., F.F., O.A., N.H., M.W., P.G.-W., A.L., R.C., and M.H.

DECLARATION OF INTERESTS

G.A.B. has received research funding from Bioverativ and Pfizer, Inc.

REFERENCES

1. Sanyal, A., Lajoie, B.R., Jain, G., and Dekker, J. (2012). The long-range interaction landscape of gene promoters. Nature *489*, 109–113. https://doi.org/10.1038/nature11279.

2. Rao, S.S.P., Huntley, M.H., Durand, N.C., Stamenova, E.K., Bochkov, I.D., Robinson, J.T., Sanborn, A.L., Machol, I., Omer, A.D., Lander, E.S., and Aiden, E.L. (2014). A 3D map of the human genome at kilobase resolution reveals principles of chromatin looping. Cell *159*, 1665–1680. https://doi.org/10.1016/j.cell.2014.11.021.

3. Brandão, H.B., Gabriele, M., and Hansen, A.S. (2021). Tracking and interpreting long-range chromatin interactions with super-resolution live-cell imaging. Curr. Opin. Cell Biol. *70*, 18–26. https://doi.org/10.1016/j.ceb.2020.11.002.

4. Galouzis, C.C., and Furlong, E.E.M. (2022). Regulating specificity in enhancer-promoter communication. Curr. Opin. Cell Biol. *75*, 102065. https://doi.org/10.1016/j.ceb.2022.01.010.

5. Bender, M.A., Bulger, M., Close, J., and Groudine, M. (2000). Beta-globin gene switching and DNase I sensitivity of the endogenous beta-globin locus in mice do not require the locus control region. Mol. Cell *5*, 387–393. https://doi.org/10.1016/s1097-2765(00)80433-5.

6. Epner, E., Reik, A., Cimbora, D., Telling, A., Bender, M.A., Fiering, S., Enver, T., Martin, D.I., Kennedy, M., Keller, G., and Groudine, M. (1998). The beta-globin LCR is not necessary for an open chromatin structure or developmentally regulated transcription of the native mouse beta-globin locus. Mol. Cell *2*, 447–455. https://doi.org/10.1016/s1097-2765(00)80144-6.

7. Carter, D., Chakalova, L., Osborne, C.S., Dai, Y.F., and Fraser, P. (2002). Long-range chromatin regulatory interactions in vivo. Nat. Genet. *32*, 623–626. https://doi.org/10.1038/ng1051.

8. Tolhuis, B., Palstra, R.J., Splinter, E., Grosveld, F., and de Laat, W. (2002). Looping and interaction between hypersensitive sites in the active beta-globin locus. Mol. Cell *10*, 1453–1465. https://doi.org/10.1016/s1097-2765(02)00781-5.

9. Drissen, R., Palstra, R.J., Gillemans, N., Splinter, E., Grosveld, F., Philipsen, S., and de Laat, W. (2004). The active spatial organization of the beta-globin locus requires the transcription factor EKLF. Genes Dev. *18*, 2485–2490. https://doi.org/10.1101/gad.317004.

10. Vakoc, C.R., Letting, D.L., Gheldof, N., Sawado, T., Bender, M.A., Groudine, M., Weiss, M.J., Dekker, J., and Blobel, G.A. (2005). Proximity among distant regulatory elements at the beta-globin locus requires GATA-1 and FOG-1. Mol. Cell *17*, 453–462. https://doi.org/10.1016/j.molcel.2004.12.028.

11. Song, S.H., Hou, C., and Dean, A. (2007). A positive role for NLI/Ldb1 in long-range beta-globin locus control region function. Mol. Cell *28*, 810–822. https://doi.org/10.1016/j.molcel.2007.09.025.

12. Patrinos, G.P., de Krom, M., de Boer, E., Langeveld, A., Imam, A.M.A., Strouboulis, J., de Laat, W., and Grosveld, F.G. (2004). Multiple interactions between regulatory regions are required to stabilize an active chromatin hub. Genes Dev. *18*, 1495–1509. https://doi.org/10.1101/gad.289704.

13. Palstra, R.J., Tolhuis, B., Splinter, E., Nijmeijer, R., Grosveld, F., and de Laat, W. (2003). The beta-globin nuclear compartment in development and erythroid differentiation. Nat. Genet. *35*, 190–194. https://doi.org/10.1038/ng1244.

14. Jackson, J.F., Odom, J.L., and Bell, W.N. (1961). Amelioration of sickle cell disease by persistent fetal hemoglobin. JAMA *177*, 867–869. https://doi.org/10.1001/jama.1961.73040380028011b.

15. Powars, D.R., Chan, L., and Schroeder, W.A. (1989). The influence of fetal hemoglobin on the clinical expression of sickle cell anemia. Ann. N. Y. Acad. Sci. *565*, 262–278. https://doi.org/10.1111/j.1749-6632.1989.tb24174.x.

16. Platt, O.S., Brambilla, D.J., Rosse, W.F., Milner, P.F., Castro, O., Steinberg, M.H., and Klug, P.P. (1994). Mortality in sickle cell disease. Life expectancy and risk factors for early death. N. Engl. J. Med. *330*, 1639–1644. https://doi.org/10.1056/NEJM199406093302303.

17. Deng, W., Lee, J., Wang, H., Miller, J., Reik, A., Gregory, P.D., Dean, A., and Blobel, G.A. (2012). Controlling long-range genomic interactions at a native locus by targeted tethering of a looping factor. Cell *149*, 1233–1244. https://doi.org/10.1016/j.cell.2012.03.051.

18. Krivega, I., and Dean, A. (2017). LDB1-mediated enhancer looping can be established independent of mediator and cohesin. Nucleic Acids Res. *45*, 8255–8268. https://doi.org/10.1093/nar/gkx433.

19. Deng, W., Rupon, J.W., Krivega, I., Breda, L., Motta, I., Jahn, K.S., Reik, A., Gregory, P.D., Rivella, S., Dean, A., and Blobel, G.A. (2014). Reactivation of developmentally silenced globin genes by forced chromatin looping. Cell *158*, 849–860.

20. Breda, L., Motta, I., Lourenco, S., Gemmo, C., Deng, W., Rupon, J.W., Abdulmalik, O.Y., Manwani, D., Blobel, G.A., and Rivella, S. (2016). Forced chromatin looping raises fetal hemoglobin in adult sickle cells to higher levels than pharmacologic inducers. Blood *128*, 1139–1143.

21. Behringer, R.R., Ryan, T.M., Palmiter, R.D., Brinster, R.L., and Townes, T.M. (1990). Human gamma- to beta-globin gene switching in transgenic mice. Genes Dev. *4*, 380–389. https://doi.org/10.1101/gad.4.3.380.

22. Gallarda, J.L., Foley, K.P., Yang, Z.Y., and Engel, J.D. (1989). The beta-globin stage selector element factor is erythroid-specific promoter/enhancer binding protein NF-E4. Genes Dev. *3*, 1845–1859. https://doi.org/10.1101/gad.3.12a.1845.

23. Bartman, C.R., Hsu, S.C., Hsiung, C.C.S., Raj, A., and Blobel, G.A. (2016). Enhancer regulation of transcriptional bursting parameters revealed by forced chromatin looping. Mol. Cell *62*, 237–247. https://doi.org/10.1016/j.molcel.2016.03.007.

24. Topfer, S.K., Feng, R., Huang, P., Ly, L.C., Martyn, G.E., Blobel, G.A., Weiss, M.J., Quinlan, K.G.R., and Crossley, M. (2022). Disrupting the adult globin promoter alleviates promoter competition and reactivates fetal globin gene expression. Blood *139*, 2107–2118. https://doi.org/10.1182/blood.2021014205.

25. Zhang, D., Lam, J., and Blobel, G.A. (2021). Engineering three-dimensional genome folding. Nat. Genet. *53*, 602–611. https://doi.org/10.1038/s41588-021-00860-9.

26. Peslak, S.A., Khandros, E., Huang, P., Lan, X., Geronimo, C.L., Grevet, J.D., Abdulmalik, O., Zhang, Z., Giardine, B.M., Keller, C.A., et al. (2020). HRI depletion cooperates with pharmacologic inducers to elevate fetal hemoglobin and reduce sickle cell formation. Blood Adv. *4*, 4560–4572. https://doi.org/10.1182/bloodadvances.2020002475.

27. Pászty, C., Brion, C.M., Manci, E., Witkowska, H.E., Stevens, M.E., Mohandas, N., and Rubin, E.M. (1997). Transgenic knockout mice with exclusively human sickle hemoglobin and sickle cell disease. Science *278*, 876–878. https://doi.org/10.1126/science.278.5339.876.

28. Uchida, N., Ferrara, F., Drysdale, C.M., Yapundich, M., Gamer, J., Nassehi, T., DiNicola, J., Shibata, Y., Wielgosz, M., Kim, Y.S., et al. (2021). Sustained fetal hemoglobin induction in vivo is achieved by BCL11A interference and coexpressed truncated erythropoietin receptor. Sci. Transl. Med. *13*, eabb0411. https://doi.org/10.1126/scitranslmed.abb0411.

29. Demirci, S., Zeng, J., Wu, Y., Uchida, N., Shen, A.H., Pellin, D., Gamer, J., Yapundich, M., Drysdale, C., Bonanno, J., et al. (2020). BCL11A enhancer–edited hematopoietic stem cells persist in rhesus monkeys without toxicity. J. Clin. Invest. *130*, 6677–6687.

30. Zhan, J., Irudayam, M.J., Nakamura, Y., Kurita, R., and Nienhuis, A.W. (2020). High level of fetal-globin reactivation by designed transcriptional activator-like effector. Blood Adv. *4*, 687–695. https://doi.org/10.1182/bloodadvances.2019000482.

31. Wienert, B., Funnell, A.P.W., Norton, L.J., Pearson, R.C.M., Wilkinson-White, L.E., Lester, K., Vadolas, J., Porteus, M.H., Matthews, J.M., Quinlan, K.G.R., and Crossley, M. (2015). Editing the genome to introduce a beneficial naturally occurring mutation associated with increased fetal globin. Nat. Commun. *6*, 7085. https://doi.org/10.1038/ncomms8085.

32. Ferster, A., Corazza, F., Vertongen, F., Bujan, W., Devalck, C., Fondu, P., Cochaux, P., Lambermont, M., Khaladji, Z., and Sariban, E. (1995). Transplanted sickle-cell disease patients with autologous bone marrow recovery after graft failure develop increased levels of fetal haemoglobin which corrects disease severity. Br. J. Haematol. *90*, 804–808. https://doi.org/10.1111/j.1365-2141.1995.tb05199.x.

33. Demirci, S., Mora, J.J.H., Yapundich, M., Drysdale, C., Gamer, J., Nassehi, T., Bonifacino, A.C., Krouse, A.E., Linde, N.S., Donahue, R.E., et al. (2019). Fetal hemoglobin and F-cell variance in mobilized CD34+ cell-transplanted rhesus monkeys. Exp. Hematol. *75*, 21–25.e1.

34. Persons, D.A., Hargrove, P.W., Allay, E.R., Hanawa, H., and Nienhuis, A.W. (2003). The degree of phenotypic correction of murine beta -thalassemia intermedia following lentiviral-mediated transfer of a human gamma-globin gene is influenced by chromosomal position effects and vector copy number. Blood *101*, 2175–2183. https://doi.org/10.1182/blood-2002-07-2211.

35. Uchida, N., Nassehi, T., Drysdale, C.M., Gamer, J., Yapundich, M., Demirci, S., Haro-Mora, J.J., Leonard, A., Hsieh, M.M., and Tisdale, J.F. (2019). High-efficiency lentiviral transduction of human CD34(+) cells in high-density culture with poloxamer and prostaglandin E2. Mol. Ther. Methods Clin. Dev. *13*, 187–196. https://doi.org/10.1016/j.omtm.2019.01.005.

36. Bauer, D.E., Kamran, S.C., Lessard, S., Xu, J., Fujiwara, Y., Lin, C., Shao, Z., Canver, M.C., Smith, E.C., Pinello, L., et al. (2013). An erythroid enhancer of BCL11A subject to genetic variation determines fetal hemoglobin level. Science *342*, 253–257. https://doi.org/10.1126/science.1242088.

37. Canver, M.C., Smith, E.C., Sher, F., Pinello, L., Sanjana, N.E., Shalem, O., Chen, D.D., Schupp, P.G., Vinjamur, D.S., Garcia, S.P., et al. (2015). BCL11A enhancer dissection by Cas9-mediated in situ saturating mutagenesis. Nature *527*, 192–197. https://doi.org/10.1038/nature15521.

38. Romero, Z., Campo-Fernandez, B., Wherley, J., Kaufman, M.L., Urbinati, F., Cooper, A.R., Hoban, M.D., Baldwin, K.M., Lumaquin, D., Wang, X., et al. (2015). The human ankyrin 1 promoter insulator sustains gene expression in a beta-globin lentiviral vector in hematopoietic stem cells. Mol. Ther. Methods Clin. Dev. *2*, 15012. https://doi.org/10.1038/mtm.2015.12.

39. Benabdallah, N.S., Williamson, I., Illingworth, R.S., Kane, L., Boyle, S., Sengupta, D., Grimes, G.R., Therizols, P., and Bickmore, W.A. (2019). Decreased enhancer-promoter proximity accompanying enhancer activation. Mol. Cell *76*, 473–484.e7. https://doi.org/10.1016/j.molcel.2019.07.038.

40. Lupiáñez, D.G., Kraft, K., Heinrich, V., Krawitz, P., Brancati, F., Klopocki, E., Horn, D., Kayserili, H., Opitz, J.M., Laxova, R., et al. (2015). Disruptions of topological chromatin domains cause pathogenic rewiring of gene-enhancer interactions. Cell *161*, 1012–1025. https://doi.org/10.1016/j.cell.2015.04.004.

41. Massaro, G., Mattar, C.N.Z., Wong, A.M.S., Sirka, E., Buckley, S.M.K., Herbert, B.R., Karlsson, S., Perocheau, D.P., Burke, D., Heales, S., et al. (2018). Fetal gene therapy for neurodegenerative disease of infants. Nat. Med. *24*, 1317–1323. https://doi.org/10.1038/s41591-018-0106-7.

42. Thompson, A.A., Walters, M.C., Kwiatkowski, J., Rasko, J.E.J., Ribeil, J.A., Hongeng, S., Magrin, E., Schiller, G.J., Payen, E., Semeraro, M., et al. (2018). Gene therapy in patients with transfusion-dependent beta-thalassemia. N. Engl. J. Med. *378*, 1479–1493. https://doi.org/10.1056/NEJMoa1705342.

43. Locatelli, F., Thompson, A.A., Kwiatkowski, J.L., Porter, J.B., Thrasher, A.J., Hongeng, S., Sauer, M.G., Thuret, I., Lal, A., Algeri, M., et al. (2022). Betibeglogene autotemcel gene therapy for non-beta(0)/beta(0) genotype beta-thalassemia. N. Engl. J. Med. *386*, 415–427. https://doi.org/10.1056/NEJMoa2113206.

44. Kanter, J., Walters, M.C., Krishnamurti, L., Mapara, M.Y., Kwiatkowski, J.L., Rifkin-Zenenberg, S., Aygun, B., Kasow, K.A., Pierciey, F.J., Jr., Bonner, M., et al. (2022). Biologic and clinical efficacy of LentiGlobin for sickle cell disease. N. Engl. J. Med. *386*, 617–628. https://doi.org/10.1056/NEJMoa2117175.

45. Long, H.K., Osterwalder, M., Welsh, I.C., Hansen, K., Davies, J.O.J., Liu, Y.E., Koska, M., Adams, A.T., Aho, R., Arora, N., et al. (2020). Loss of extreme long-range enhancers in human neural crest drives a craniofacial disorder. Cell Stem Cell *27*, 765–783.e14. https://doi.org/10.1016/j.stem.2020.09.001.

46. Claringbould, A., and Zaugg, J.B. (2021). Enhancers in disease: molecular basis and emerging treatment strategies. Trends Mol. Med. *27*, 1060–1073. https://doi.org/10.1016/j.molmed.2021.07.012.

47. Esrick, E.B., Lehmann, L.E., Biffi, A., Achebe, M., Brendel, C., Ciuculescu, M.F., Daley, H., MacKinnon, B., Morris, E., Federico, A., et al. (2021). Post-transcriptional genetic silencing of BCL11A to treat sickle cell disease. N. Engl. J. Med. *384*, 205–215. https://doi.org/10.1056/NEJMoa2029392.

48. Frangoul, H., Ho, T.W., and Corbacioglu, S. (2021). CRISPR-Cas9 gene editing for sickle cell disease and beta-thalassemia. N. Engl. J. Med. *384*, e91. https://doi.org/10.1056/NEJMc2103481.

49. Weber, L., Frati, G., Felix, T., Hardouin, G., Casini, A., Wollenschlaeger, C., Meneghini, V., Masson, C., De Cian, A., Chalumeau, A., et al. (2020). Editing a gamma-globin repressor binding site restores fetal hemoglobin synthesis and corrects the sickle cell disease phenotype. Sci. Adv. *6*, eaay9392. https://doi.org/10.1126/sciadv.aay9392.

50. Ravi, N.S., Wienert, B., Wyman, S.K., Bell, H.W., George, A., Mahalingam, G., Vu, J.T., Prasad, K., Bandlamudi, B.P., Devaraju, N., et al. (2022). Identification of novel HPFH-like mutations by CRISPR base editing that elevate the expression of fetal hemoglobin. Elife *11*, e65421. https://doi.org/10.7554/eLife.65421.

51. Newby, G.A., Yen, J.S., Woodard, K.J., Mayuranathan, T., Lazzarotto, C.R., Li, Y., Sheppard-Tillman, H., Porter, S.N., Yao, Y., Mayberry, K., et al. (2021). Base editing of haematopoietic stem cells rescues sickle cell disease in mice. Nature *595*, 295–302. https://doi.org/10.1038/s41586-021-03609-w.

52. Grevet, J.D., Lan, X., Hamagami, N., Edwards, C.R., Sankaranarayanan, L., Ji, X., Bhardwaj, S.K., Face, C.J., Posocco, D.F., Abdulmalik, O., et al. (2018). Domain-focused CRISPR screen identifies HRI as a fetal hemoglobin regulator in human erythroid cells. Science *361*, 285–290. https://doi.org/10.1126/science.aao0932.

53. Gräslund, T., Li, X., Magnenat, L., Popkov, M., and Barbas, C.F., 3rd (2005). Exploring strategies for the design of artificial transcription factors: targeting sites proximal to known regulatory regions for the induction of gamma-globin expression and the treatment of sickle cell disease. J. Biol. Chem. *280*, 3707–3714. https://doi.org/10.1074/jbc.M406809200.

54. Huang, P., Peslak, S.A., Lan, X., Khandros, E., Yano, J.A., Sharma, M., Keller, C.A., Giardine, B., Qin, K., Abdulmalik, O., et al. (2020). The HRI-regulated transcription factor ATF4 activates BCL11A transcription to silence fetal hemoglobin expression. Blood *135*, 2121–2132. https://doi.org/10.1182/blood.2020005301.

55. Khandros, E., Huang, P., Peslak, S.A., Sharma, M., Abdulmalik, O., Giardine, B.M., Zhang, Z., Keller, C.A., Hardison, R.C., and Blobel, G.A. (2020). Understanding heterogeneity of fetal hemoglobin induction through comparative analysis of F and A erythroblasts. Blood *135*, 1957–1968. https://doi.org/10.1182/blood.2020005058.

56. Modlich, U., Schambach, A., Li, Z., and Schiedlmeier, B. (2009). Murine hematopoietic stem cell transduction using retroviral vectors. Methods Mol. Biol. *506*, 23–31. https://doi.org/10.1007/978-1-59745-409-4_3.

57. Abdulmalik, O., Safo, M.K., Chen, Q., Yang, J., Brugnara, C., Ohene-Frempong, K., Abraham, D.J., and Asakura, T. (2005). 5-hydroxymethyl-2-furfural modifies intracellular sickle haemoglobin and inhibits sickling of red blood cells. Br. J. Haematol. *128*, 552–561. https://doi.org/10.1111/j.1365-2141.2004.05332.x.

58. Jang, Y., Kim, Y.S., Wielgosz, M.M., Ferrara, F., Ma, Z., Condori, J., Palmer, L.E., Zhao, X., Kang, G., Rawlings, D.J., et al. (2020). Optimizing lentiviral vector transduction of hematopoietic stem cells for gene therapy. Gene Ther. *27*, 545–556. https://doi.org/10.1038/s41434-020-0150-z.

59. Bauler, M., Roberts, J.K., Wu, C.C., Fan, B., Ferrara, F., Yip, B.H., Diao, S., Kim, Y.I., Moore, J., Zhou, S., et al. (2020). Production of lentiviral vectors using suspension cells grown in serum-free media. Mol. Ther. Methods Clin. Dev. *17*, 58–68. https://doi.org/10.1016/j.omtm.2019.11.011.

60. Uchida, N., Li, L., Nassehi, T., Drysdale, C.M., Yapundich, M., Gamer, J., Haro-Mora, J.J., Demirci, S., Leonard, A., Bonifacino, A.C., et al. (2021). Preclinical evaluation for engraftment of CD34(+) cells gene-edited at the sickle cell disease locus in xenograft mouse and non-human primate models. Cell Rep. Med. *2*, 100247. https://doi.org/10.1016/j.xcrm.2021.100247.

61. Demirci, S., Bhardwaj, S.K., Uchida, N., Haro-Mora, J.J., Ryu, B., Blobel, G.A., and Tisdale, J.F. (2018). Robust erythroid differentiation system for rhesus hematopoietic progenitor cells allowing preclinical screening of genetic treatment strategies for the hemoglobinopathies. Cytotherapy *20*, 1278–1287.

62. Uchida, N., Evans, M.E., Hsieh, M.M., Bonifacino, A.C., Krouse, A.E., Metzger, M.E., Sellers, S.E., Dunbar, C.E., Donahue, R.E., and Tisdale, J.F. (2013). Integration-specific in vitro evaluation of lentivirally transduced rhesus CD34+ cells correlates with in vivo vector copy number. Mol. Ther. Nucleic Acids *2*, e122.

OMTN, Volume *31*

Supplemental information

Forced enhancer-promoter rewiring

to alter gene expression in animal models

Scott A. Peslak, Selami Demirci, Vemika Chandra, Byoung Ryu, Saurabh K. Bhardwaj, Jing Jiang, Jeremy W. Rupon, Robert E. Throm, Naoya Uchida, Alexis Leonard, Khaled Essawi, Aylin C. Bonifacino, Allen E. Krouse, Nathaniel S. Linde, Robert E. Donahue, Francesca Ferrara, Matthew Wielgosz, Osheiza Abdulmalik, Nicole Hamagami, Paula Germino-Watnick, Anh Le, Rebecca Chu, Malikiya Hinds, Mitchell J. Weiss, Wei Tong, John F. Tisdale, and Gerd A. Blobel

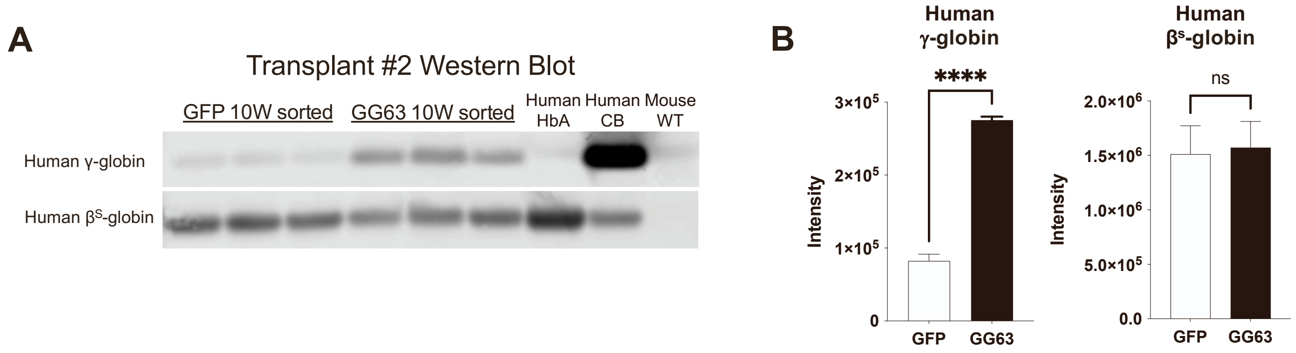

Figure S1. Forced ZF-Ldb1 chromatin looping in the BERK mouse model drives in vivo induction of γ-globin expression. (A) Western blot of an independent transplantation experiment utilizing the GG63-Ldb1-GFP construct showed consistent, sustained in vivo expression of γ-globin as compared to GFP control. (B) Quantification of γ-globin and β^S-globin proteins. N=3 transplanted mice for each condition. Statistical analysis done using Student's t-test. Error bars represent standard deviation; ns, not significant; ****, $p<0.0001$. CB, cord blood; WT, wild-type.

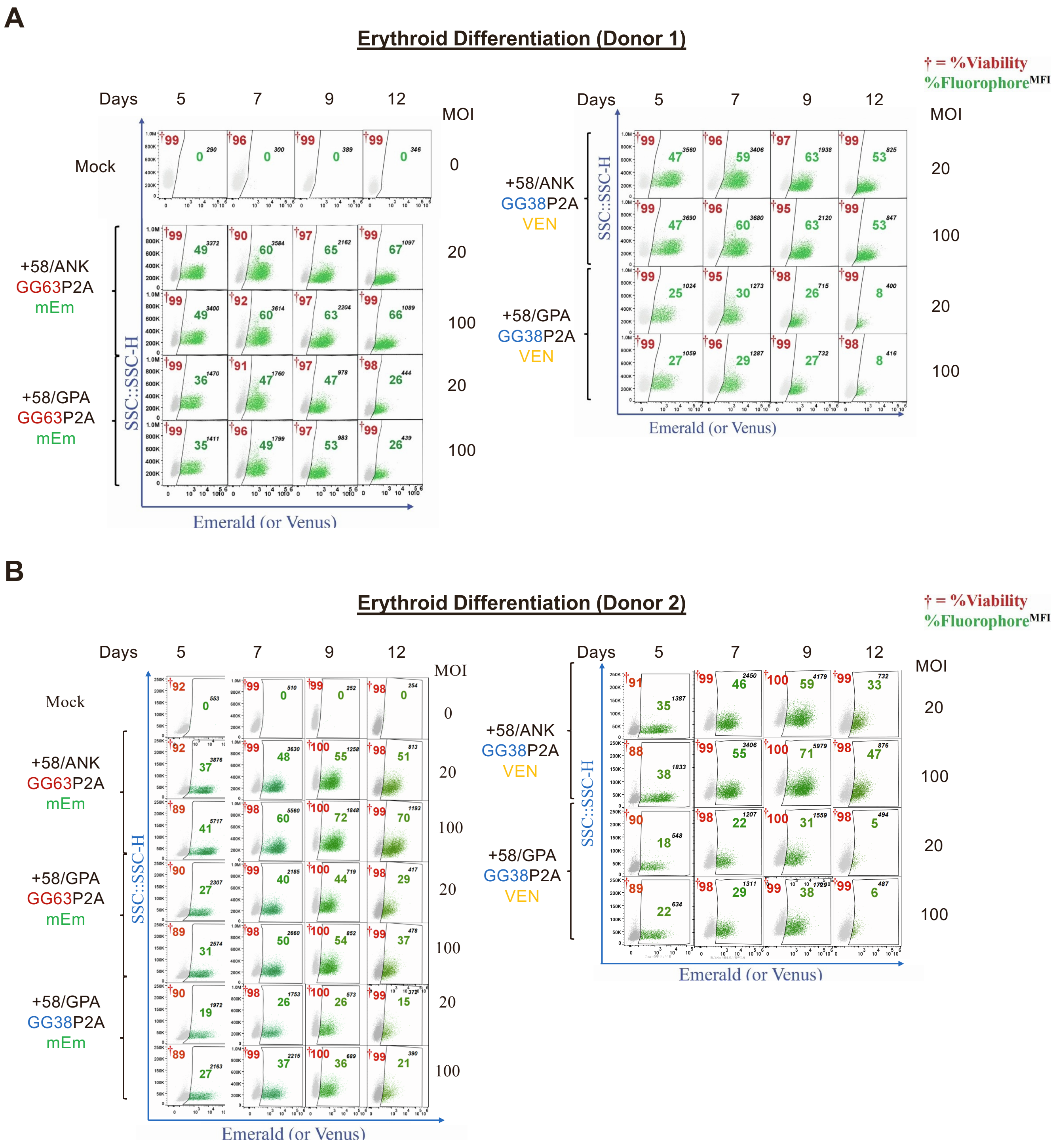

Figure S2. Transgene expression and viability analysis of transduced rhesus CD34^{+} cells. Data shown at days 5, 7, 9, and 12 of in vitro erythroid culture for donor #1 (A) and donor #2 (B). Cellular viability is shown in red (% viability). Vector expression (measured by percent mEmerald or Venus by flow cytometric analysis) is quantified in green, with mean fluorescence intensity (MFI) as superscript (%Fluorophore^{MFI}). N=2 independent donors.

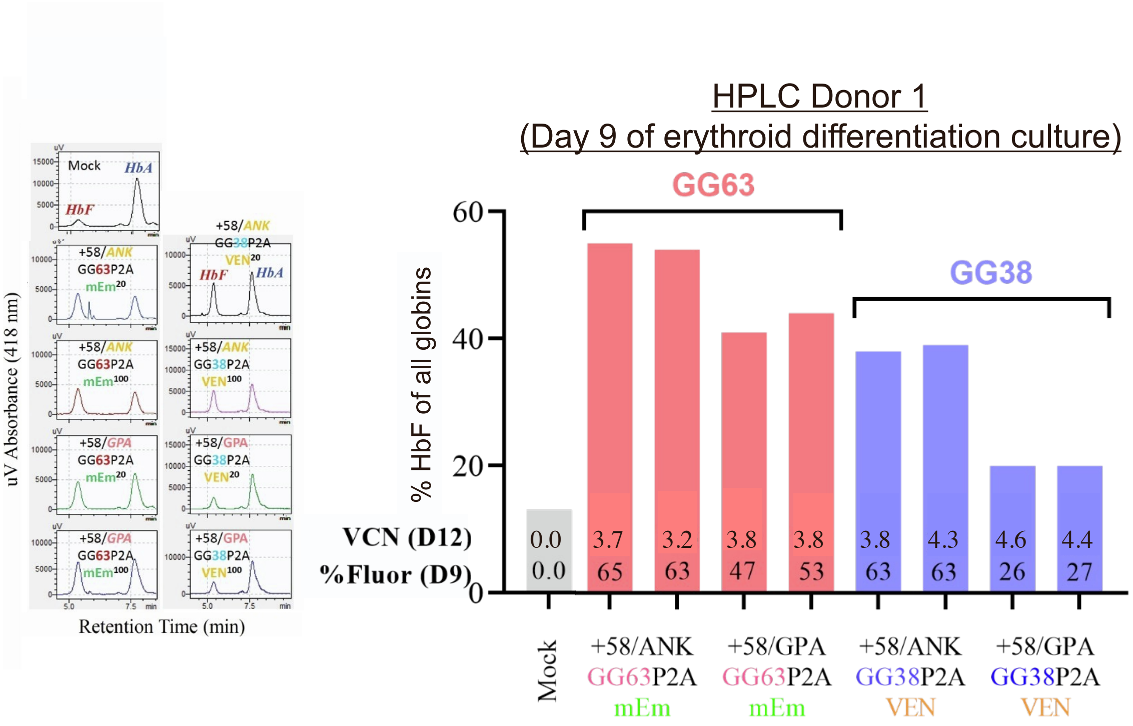

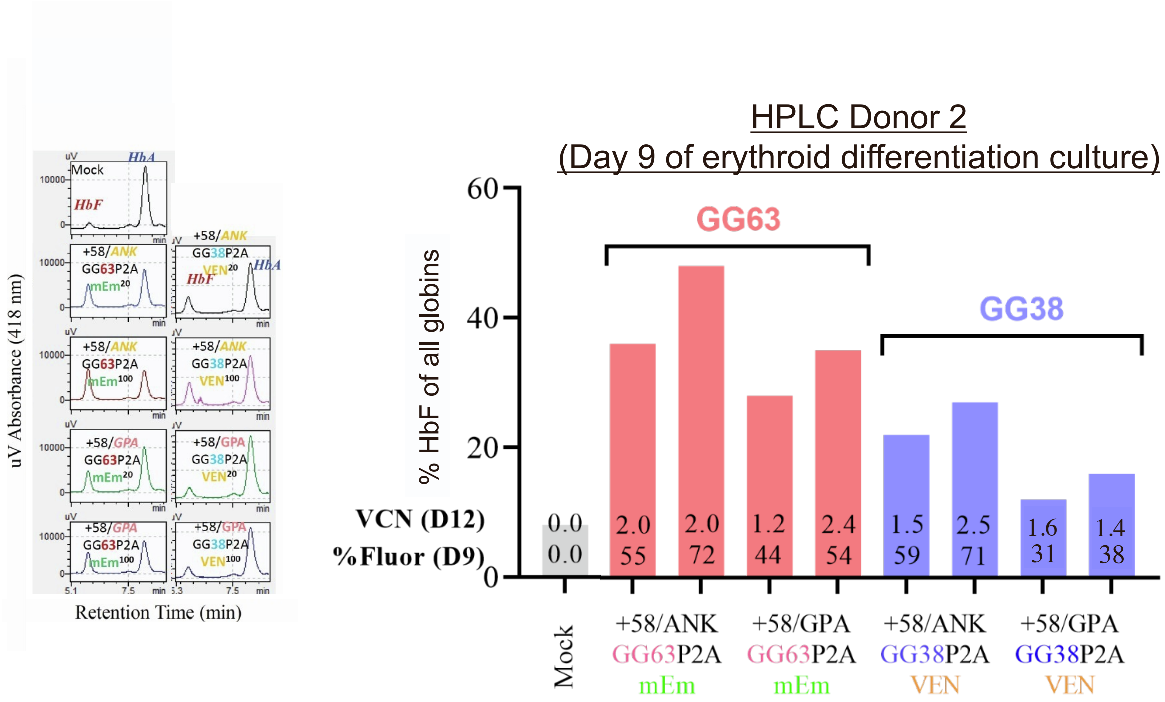

Figure S3. HPLC analysis following transduction of optimized zinc finger constructs in two independent human CD34⁺ donor cells at day 9 of erythroid differentiation culture. HPLC elution tracings (left) and quantification of HPLC peaks (right) illustrate high levels of HbF induction. Vector copy number (VCN D12) and percent Venus/mEmerald positivity (%Fluor D9) are displayed for each vector tested. N=2 independent donors.

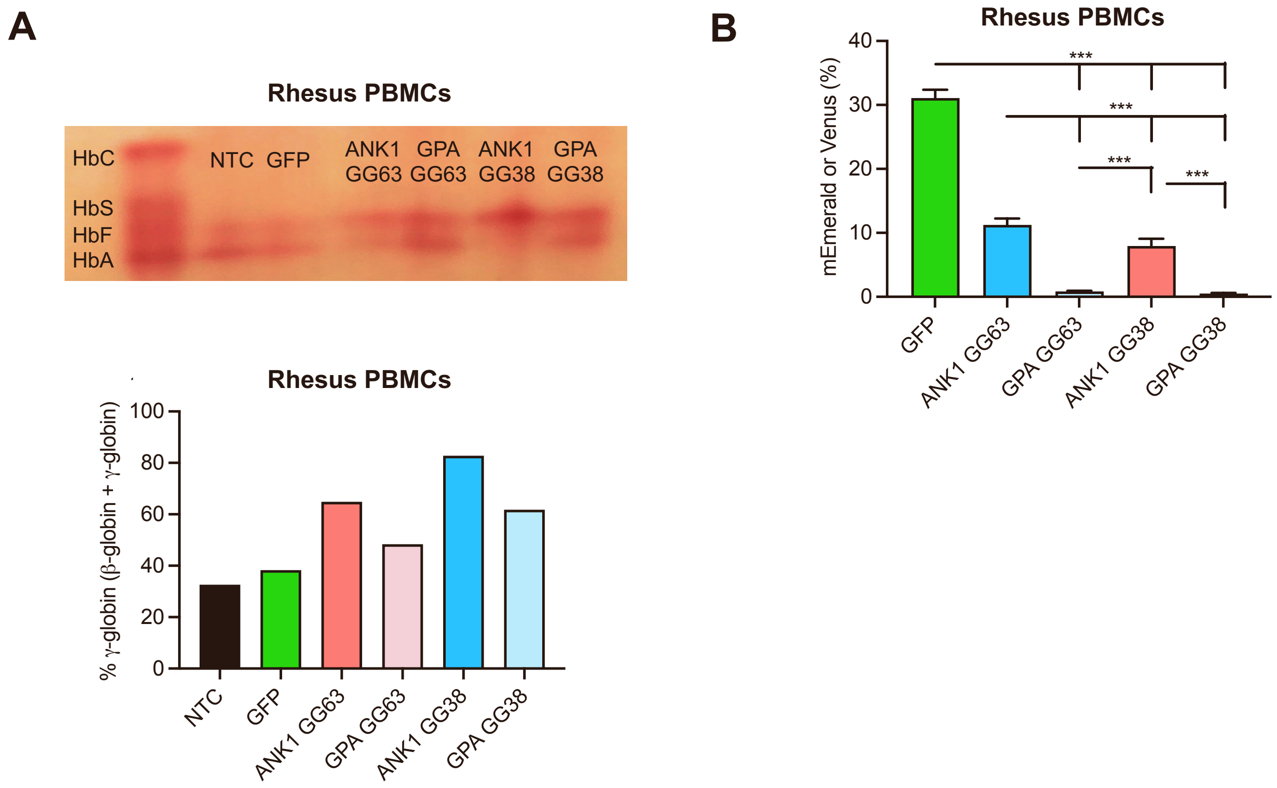

Figure S4. Robust ex vivo γ-globin induction in rhesus progenitor cells transduced with optimized zinc finger constructs. (A) Hemoglobin electrophoresis (top) and RP-HPLC (bottom) in differentiated rhesus PBMCs, N=1. (B) Flow cytometry analysis of mEmerald or Venus vector expression in differentiated rhesus PBMCs. Cells were transduced with the ZF lentiviruses (MOI=50 for CD34$^+$ and MOI=10 for PBMCs) at high cell density (2×10^6cells/mL) in XVIVO-10 media + SFT (100 ng/mL each), N=3. Statistical analyses done using one-way ANOVA analysis. Error bars represent standard deviation; ***, $p<0.001$. HbF and flow cytometric assays were performed on unsorted bulk erythroid cells. HbC, hemoglobin C; HbS, hemoglobin S/sickle hemoglobin; HbF, hemoglobin F/fetal hemoglobin; HbA, hemoglobin A/adult hemoglobin.

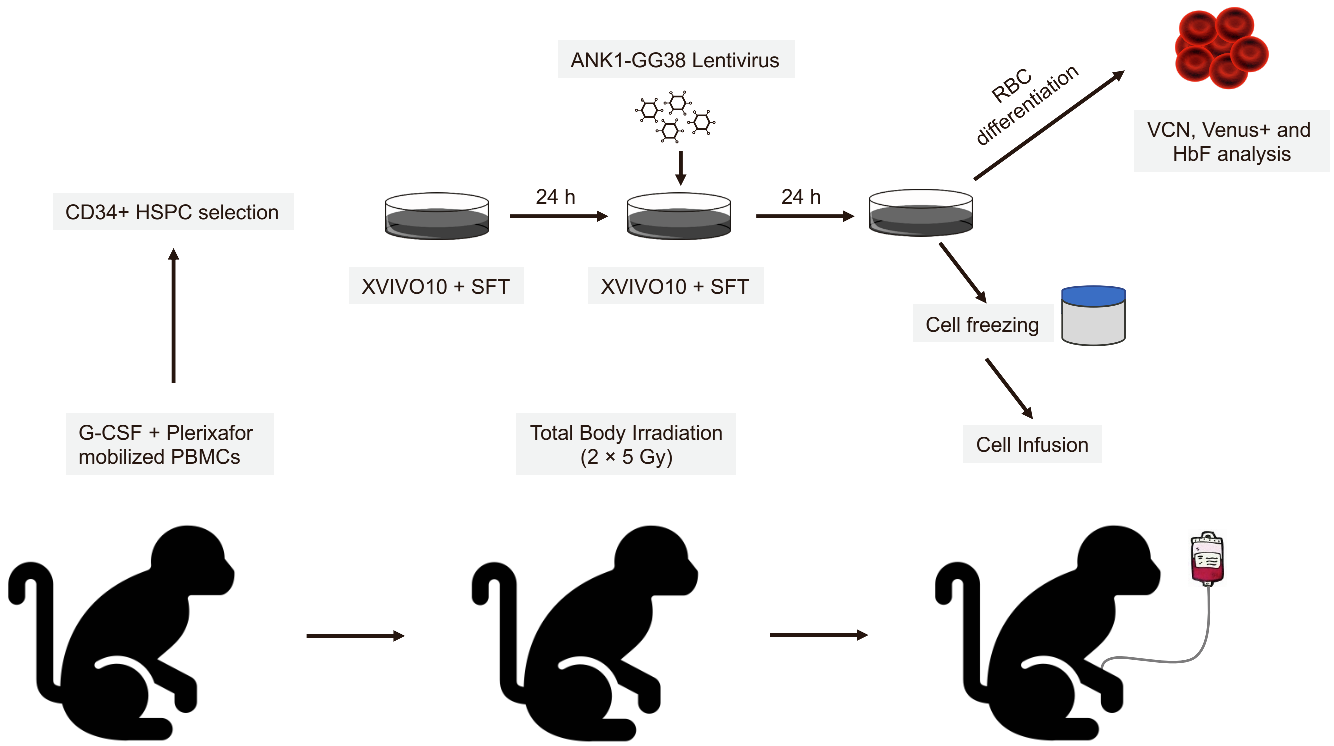

Figure S5. Representative illustration for in vivo experimental design for rhesus macaque studies. A small portion of the transduced $CD34^+$ hematopoietic stem and progenitor cells (HSPCs) were used for red blood cell (RBC) differentiation to confirm vector copy number (VCN), reporter gene (Venus), and fetal hemoglobin (HbF) induction before cell infusion. SFT, Stem cell factor, Flt-3, TPO.

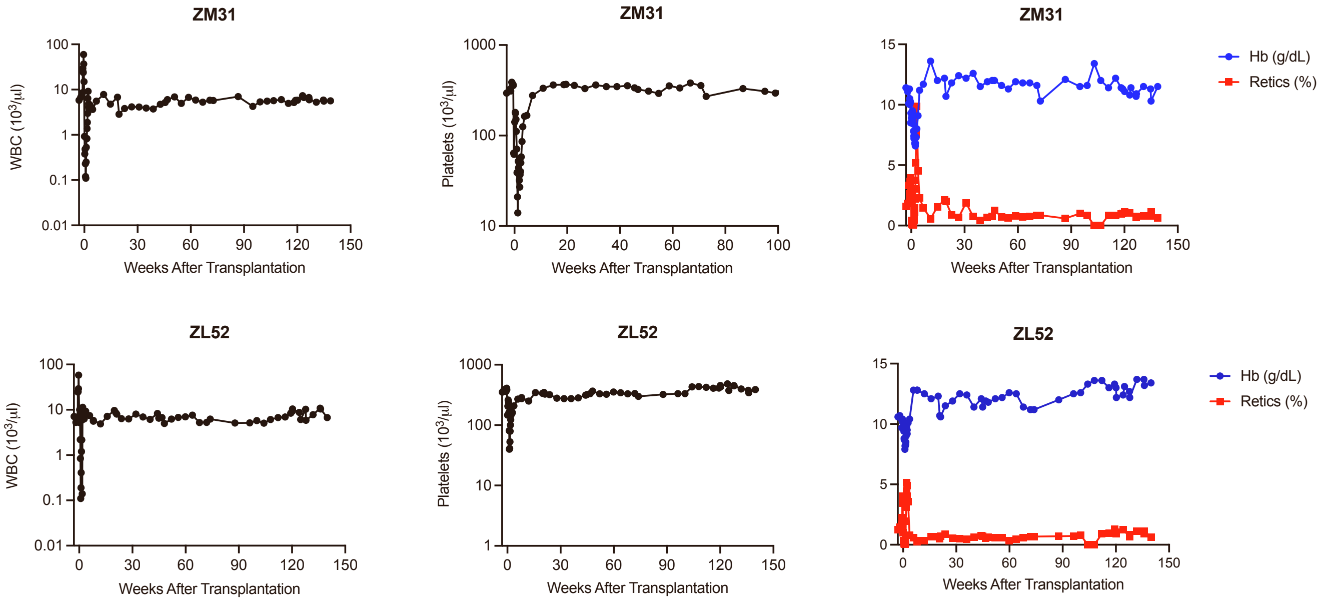

Figure S6. Peripheral blood parameters in transplanted rhesus macaques. WBC, white blood cells; Hb, hemoglobin; Retics, reticulocyte percentage. N=2 independent transplanted rhesus macaques.

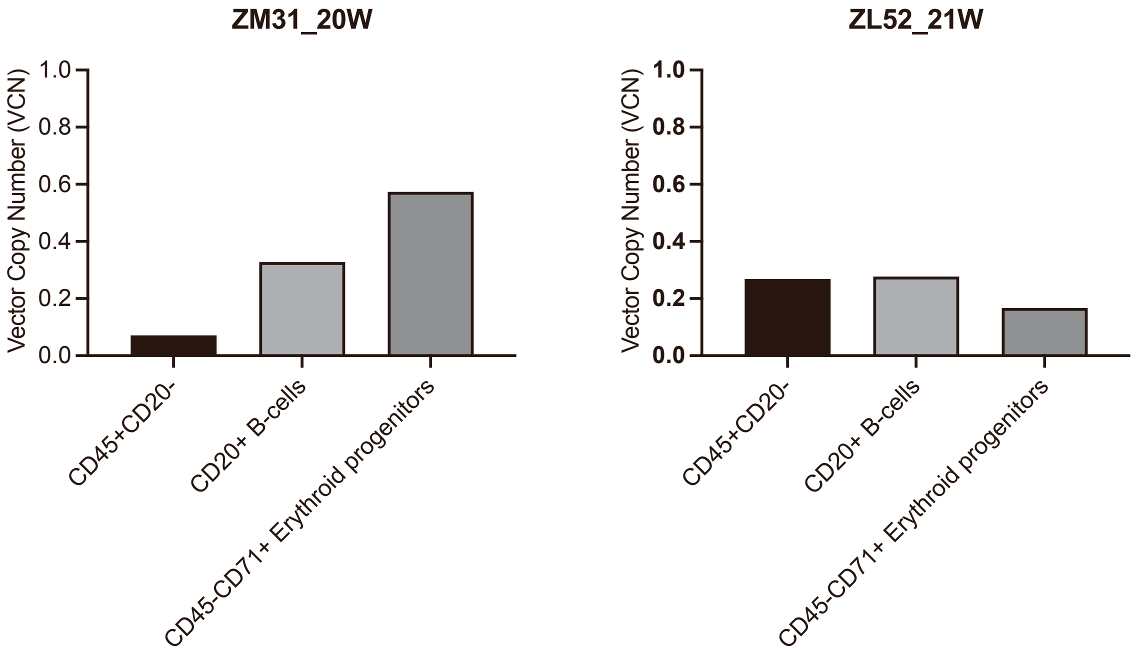

Figure S7. **Vector copy number analysis of transplanted macaques**. Vector copy number (VCN) was determined for CD20^{+} B-cells, CD45^{-}CD71^{+} erythroid progenitors, and all other CD45^{+} cells (CD45^{+}CD20^{-}). N=2 independent transplanted rhesus macaques.

Table S1. Differentially expressed genes (all DEG, N=33) by RNA-seq analysis following forced chromatin looping with GG38 ZF construct. Differentially expressed genes (defined as absolute fold change greater than 1.5 and false discovery rate (FDR) less than 0.05) between GFP-only control and GG38 treated samples. Two biologically independent replicates were performed, and log_2 fold change, p-value, and FDR are displayed for each differentially expressed gene.

Gene Name	Log_2 Fold Change	p-value	FDR
HBG2	3.391192224	1.03871850193996E-64	1.05637671647294E-61
HBG1	1.758872384	4.92265544578652E-18	2.50317029418245E-15
AC104389.6	1.887867842	3.68731774982421E-16	1.25000071719041E-13
RN7SL1	11.45843448	1.71914607021391E-14	4.37092888351887E-12
ENSG00000206028	-2.015993142	7.40936805573879E-08	1.50706546253727E-05
CLK3	-0.962098867	3.39970517514562E-07	5.76250027187182E-05
AC106788.3	2.308955752	7.94076631485196E-06	0.001150774
LMOD1	4.720262133	9.05230313845142E-06	0.001150774
HBD	-1.244358975	1.68169128586907E-05	0.001900311
HBB	-0.867365146	2.6758393268163E-05	0.002537381
ENSG00000265401	-0.947692982	2.74446298830256E-05	0.002537381
PAQR3	-0.849642798	4.00466594326636E-05	0.003393954
WDR5B-DT	-0.972440856	5.22273379075885E-05	0.004085785
DDIT4-AS1:3	0.96084767	0.00010045	0.006816809
CCNDBP1	-0.847377884	0.000100543	0.006816809
COG1	-0.866773776	0.000156075	0.009920547
ENSG00000266642	-0.793092242	0.000267332	0.015992741
PTOV1-AS1	-1.131813167	0.000297157	0.016789377
RNF141	-1.848561334	0.000344087	0.01841772
GMPPB	-0.89928835	0.000497951	0.024385615
ENSG00000269984	-1.023123598	0.000503538	0.024385615
ABALON	-0.804037381	0.000690933	0.031627761
TAF10	-0.943823154	0.000715279	0.031627761
IL16	-1.402787456	0.000772927	0.03275279
ENSG00000279159	-0.758754634	0.000819498	0.033337167
GADD45GIP1	-0.738302076	0.000911435	0.035651132
STEAP3-AS1	-1.067377965	0.001197563	0.042979078
TMEM50A	-1.207911739	0.001214041	0.042979078
BEST3	-0.929417047	0.001225559	0.042979078
LARP1	-1.11365211	0.001333373	0.044527676
UBE2N	-0.686687731	0.00139675	0.044527676
PSMG4	-0.746330927	0.001401067	0.044527676
ENSG00000239665	-1.009267904	0.001490096	0.04592205

Table S2. Differentially expressed genes (all DEG, N=17) by RNA-seq analysis following forced chromatin looping with GG63 ZF construct. Differentially expressed genes (defined as absolute fold change greater than 1.5 and false discovery rate (FDR) less than 0.05) between GFP-only control and GG63 treated samples. Two biologically independent replicates were performed, and $\log_2$ fold change, p-value, and FDR are displayed for each differentially expressed gene.

Gene Name	$\log_2$ Fold Change	p-value	FDR
HBG2	3.088062801	1.17832459239214E-66	5.19051982948736E-63
HBG1	1.525015142	1.58389463268251E-15	3.48852792848322E-12
AC104389.6	1.655987469	1.18598645747015E-13	1.74142344838533E-10
HBB	-1.090908831	6.44017015221578E-09	7.09223738012763E-06
IGF2	9.068903479	5.32468221730751E-08	4.41377700565219E-05
ENSG00000260121	3.114699433	6.0119550587771E-08	4.41377700565219E-05
ABLIM1	3.760551318	1.89172027210083E-07	0.000119043
HBD	-1.842744945	1.21592899423866E-06	0.000669521
ENSG00000262211	-1.117812263	2.03091080037609E-06	0.000994018
NUDT4B	8.328044636	2.95126308678451E-06	0.001300031
CLMN	4.491272925	5.11289316647602E-06	0.002047481
CLK3	-0.668917158	5.09039212144048E-05	0.018685981
GALNT7	-1.272691299	5.899598873941E-05	0.019990564
MSN	4.763017708	9.58764175311498E-05	0.03016683
GADD45GIP1	-0.828050474	0.000140142	0.041154893
ENSG00000266642	-0.838953987	0.000152756	0.042055549
CERKL	-0.800806786	0.000191864	0.04971524

Table S3. Differentially expressed genes (top 100 by FDR, total DEG=409) by RNA-seq analysis following forced chromatin looping with GG52 ZF construct. Differentially expressed genes (defined as absolute fold change greater than 1.5 and false discovery rate (FDR) less than 0.05) between GFP-only control and GG52 treated samples. Top 100 results sorted by FDR are displayed, total N=409 differentially expressed genes. Two biologically independent replicates were performed, and log_2 fold change, p-value, and FDR are displayed for each differentially expressed gene.

Gene Name	Log_2 Fold Change	p-value	FDR
CLK3	-2.432215887	5.14501390782645E-54	2.2056674622852E-50
HBG2	2.794534378	1.19282894916777E-53	2.55682885254112E-50
ENSG00000266642	-2.094450993	1.31914672947036E-27	1.88506067641314E-24
HEMGN	-1.985836414	8.42300079850547E-24	9.02735110579824E-21
FAM149B1	-1.479476299	7.12683537354198E-22	6.11054864927489E-19
GMPPB	-1.986100799	2.54702507988224E-21	1.81984941957586E-18
GGCX	1.998808056	5.14827458097758E-19	3.15295044695013E-16
HBG1	1.451239885	4.17923259898292E-17	2.23954626897997E-14
ENSG00000272540	-1.292472555	2.18213162352073E-16	1.0344070559669E-13
FBXW8	-2.862197616	2.41289259614392E-16	1.0344070559669E-13
STEAP3-AS1	-2.095233062	3.09379256095256E-14	1.16578653972603E-11
CERKL	-1.393661481	3.26322334422962E-14	1.16578653972603E-11
AC104389.6	1.618796832	6.90027640169904E-14	2.21502359841522E-11
FTL	1.759940212	7.23357368271825E-14	2.21502359841522E-11
ENSG00000266872	2.905299274	9.01855388621453E-14	2.57750270068011E-11
ENSG00000265401	-1.362096235	3.08096303431498E-13	8.2550553300677E-11
ENSG00000254680	-2.142572587	4.71605508199926E-13	1.18927812567828E-10
HK1	-1.874265815	1.43178626081164E-12	3.2438332060107E-10
ENSG00000247131	-2.241311901	1.4376680875718E-12	3.2438332060107E-10
SPATA9	-1.323181237	2.39508977517318E-12	5.13387493308371E-10
HSPA5	2.108172267	2.59831228391208E-12	5.30426893387196E-10
PIGF	5.08835732	5.31655915266376E-12	1.03600404943043E-09
ENSG00000261684	1.440104194	7.17110285448898E-12	1.33663121466062E-09
ENSG00000285103	2.897394706	8.45054599555291E-12	1.50947877845564E-09
PRR29	3.000667646	9.73499132308371E-12	1.66935631208239E-09
TMPO-AS1	-1.791930918	1.21261674222299E-11	1.99941845150382E-09
H2BC15	-1.173938345	4.20710304703006E-11	6.67994472689551E-09
HOOK2	-2.373376925	5.24116429791466E-11	8.02459690898576E-09
YIPF2	-1.968112256	8.19931778021217E-11	1.21208535599205E-08
TFRC	-1.226632033	9.86782669779182E-11	1.41011243511445E-08
HSP90AB1	1.858908199	1.20811037890345E-10	1.67069974011583E-08
IST1	-1.420613869	1.27714718212911E-10	1.7109781155586E-08
NEAT1	1.369457365	1.40791397251871E-10	1.82900824248113E-08
S100A6	3.426210015	2.22037624904285E-10	2.79963322930785E-08
ENSG00000265784	2.019372481	2.42630640843238E-10	2.97187873512846E-08
LMNA	2.122976882	3.20378179355004E-10	3.81517015248584E-08

**COG1**	-1.211016937	3.42137678287137E-10	3.96417358599177E-08
**ENSG00000258017**	-0.931803008	3.60162968815807E-10	4.06320696661412E-08
**MAT2A**	2.146230958	6.14654950633595E-10	6.75647634196467E-08
**ENSG00000253389**	-1.325364487	1.72878339187919E-09	1.85282360024652E-07
**NECAB1**	-1.149569167	2.26377261722549E-09	2.36702273415749E-07
**GALNT7**	-1.383013007	3.23218369484442E-09	3.29913607138049E-07
**BEST3**	-1.341359684	3.81775675245552E-09	3.8062146971574E-07
**SIK2**	-1.608240528	4.6137811867478E-09	4.49529089717905E-07
**APLNR**	3.943399456	6.9390261547071E-09	6.61057891671763E-07
**OR52A5**	-3.368108937	7.64987559924463E-09	7.12935145520907E-07
**TMEM120B**	-3.350839786	1.03789975780793E-08	9.46697076962251E-07
**PPP1R13B**	-1.651903258	1.06362219822436E-08	9.49947575789131E-07
**ENSG00000279551**	-1.797627505	1.1477855907111E-08	1.00419527089357E-06
**ENSG00000265263**	-1.230239236	1.43926389589559E-08	1.23402486434088E-06
**ENSG00000267062**	-1.089797422	1.77932463680139E-08	1.49567935646423E-06
**CAT**	-1.400720853	1.86997736100669E-08	1.54165248973763E-06
**DNAJB12**	2.341018477	2.38060116649923E-08	1.92559192467588E-06
**ENSG00000262791**	-2.124481215	2.57242882587081E-08	2.04222266231633E-06
**TSTD2**	-1.042460388	3.90932248060542E-08	3.04713917715554E-06
**FARP1**	-0.87558818	4.03168041299383E-08	3.08639534473296E-06
**MT-ND2**	1.487581595	4.68346964350177E-08	3.52246216871791E-06
**GYS1**	2.643534403	5.45771125114104E-08	4.034001402352E-06
**ENSG00000257607**	-1.304886578	5.90071146894155E-08	4.28751696056821E-06
**ENSG00000280416**	2.305103819	6.01264494482016E-08	4.296034813074E-06
**ENSG00000285600**	-1.105966992	6.60233606843351E-08	4.64003520088106E-06
**ERAL1**	-1.695640537	8.52063647077359E-08	5.89160783067845E-06
**CLCC1**	-1.070144919	9.93627292182861E-08	6.76139714537766E-06
**WDR73**	-1.361592893	1.02986321602645E-07	6.87985582652496E-06
**DDIT4-AS1:3**	0.967846836	1.04313186079805E-07	6.87985582652496E-06
**PDP2**	8.404999222	1.30309910688657E-07	8.46422101700411E-06
**LCORL**	-1.133234731	1.34671793542554E-07	8.61698475995419E-06
**INTS8**	-1.248248627	1.53055130336387E-07	9.56434196481623E-06
**PRC1-AS1**	-1.161370769	1.53939723716426E-07	9.56434196481623E-06
**STARD5**	-8.312250188	1.66914167694673E-07	1.02223005272437E-05
**ENSG00000279159**	-0.936822329	2.01963173358758E-07	1.21945932984366E-05
**MT-ATP6**	1.74044102	2.07548089903493E-07	1.23577591863371E-05
**OSBPL11**	2.820540008	2.21771549596352E-07	1.30237620975282E-05
**OR10Z1**	-0.861468838	2.3049770368742E-07	1.33532926447023E-05
**STXBP5**	3.898067589	2.40697126332593E-07	1.3758247741171E-05
**CERS6-AS1**	2.555660592	3.26838242995559E-07	1.84362572068679E-05
**ENSG00000251600**	-1.266984569	4.43471804704887E-07	2.46904367112968E-05
**GADD45GIP1**	-1.089370909	4.59576273954568E-07	2.52590190569645E-05
**ENSG00000272418**	-1.752388205	4.98588668527815E-07	2.70563243288449E-05
**AGK**	2.62455101	5.30862067877239E-07	2.84475710623715E-05
**ENSG00000282542**	-1.14572389	5.61956240855607E-07	2.97420543771356E-05
**BTG1-DT**	1.281131349	7.421411416597E-07	3.87995009060382E-05
**ENSG00000238273**	-8.030862939	7.66442316030688E-07	3.95872073352236E-05
**PLEKHG3**	-1.396937984	7.95494497679704E-07	4.05986298994392E-05
**FRMD5**	-1.886777857	8.12017238489247E-07	4.09543282518047E-05
**MALAT1**	0.743623075	8.42079478056495E-07	4.19766828189325E-05

**ATG14**	-1.444069684	8.62482221250726E-07	4.24995549712858E-05
**ENSG00000228748**	1.911656255	8.85223945913177E-07	4.31244892742022E-05
**ARID3B**	-2.097838188	9.7829394788114E-07	4.71229905007466E-05
**HBB**	-0.886178508	1.0075573767192E-06	4.79933163777246E-05
**MAP3K4**	4.294729186	1.02649813700001E-06	4.83582144320772E-05
**MIR1302-9HG**	-1.109902288	1.14525584150282E-06	5.33664325274194E-05
**ABI3**	-2.815626944	1.29457789036755E-06	5.9675864688233E-05
**ENSG00000239665**	-1.145742043	1.58733311890088E-06	7.2392522135405E-05
**LMOD1**	4.411125545	1.62811880884782E-06	7.34330618341621E-05
**DIS3**	-1.136237265	1.64440726290636E-06	7.34330618341621E-05
**TAF10**	-1.288007722	1.67234935615673E-06	7.39109452561226E-05
**ENSG00000259248**	1.465045418	1.70662532688518E-06	7.46561507791507E-05
**ANXA1**	7.26581351	1.74639463428412E-06	7.56241797694547E-05
**ENSG00000206028**	-2.545660438	1.80122406151495E-06	7.72184755171457E-05

Table S4. Differentially expressed genes (top 100 by FDR, total DEG=677) by RNA-seq analysis following forced chromatin looping with GG1 ZF construct. Differentially expressed genes (defined as absolute fold change greater than 1.5 and false discovery rate (FDR) less than 0.05) between GFP-only control and GG1 treated samples. Top 100 results sorted by adjusted P value are displayed, total N=677 differentially expressed genes. Two biologically independent replicates were performed, and log_2 fold change, p-value, and FDR are displayed for each differentially expressed gene.

Gene Name	Log_2 Fold Change	p-value	FDR
CLK3	-2.727882	4.33237E-62	3.9078E-58
ENSG00000206028	-6.6416247	1.10015E-50	4.96168E-47
HBG2	2.89445347	1.74479E-49	5.24601E-46
CERKL	-2.664109	1.88434E-46	4.24918E-43
ENSG00000262211	-2.6360942	3.22787E-32	5.82308E-29
HBD	-2.8674076	3.40427E-31	5.11776E-28
STEAP3-AS1	-3.4032636	4.89392E-31	6.30616E-28
FAM149B1	-1.748942	5.3688E-27	6.05332E-24
GGCX	2.28056268	4.16063E-26	4.16987E-23
TCHP	2.90312058	2.94641E-25	2.65766E-22
ENSG00000269926	1.74724404	1.85639E-22	1.52224E-19
ENSG00000265401	-2.017093	6.07293E-22	4.56482E-19
FAM83A-AS1	3.03841883	1.49474E-21	1.03712E-18
TKT	4.02062249	3.21024E-19	2.06831E-16
HBG1	1.52098425	3.63148E-19	2.18373E-16
BEST3	-2.0061645	7.0176E-18	3.95617E-15
YIPF2	-2.1085721	1.00445E-16	5.32948E-14
ENSG00000251095	-2.2396276	1.36542E-16	6.8423E-14
GMPPB	-1.6288886	2.45552E-16	1.16573E-13
ENSG00000247324	-3.3226552	2.40442E-15	1.08439E-12
IST1	-1.6858456	5.63388E-15	2.41988E-12
FAM13A-AS1	-2.7404672	8.14426E-15	3.33914E-12
ENSG00000247131	-2.3657665	2.05591E-14	8.06275E-12
SNCA	-2.6385337	3.59215E-14	1.35005E-11
DMAC2L	-1.9300216	4.8047E-14	1.73354E-11
FAM129A	1.84287041	2.58197E-13	8.95745E-11
CCDC144CP	2.9088118	4.0613E-13	1.31888E-10
MAT2A	2.33435158	4.09408E-13	1.31888E-10
TAX1BP1	-1.6173907	7.57117E-13	2.35489E-10
JMJD1C	-2.1215031	9.3127E-13	2.80002E-10
TMCC2	-2.4710061	1.1074E-12	3.22218E-10
ENSG00000280064	2.12617256	1.15923E-12	3.26757E-10
UBB	-1.409654	1.38897E-12	3.69512E-10
RNF141	-2.7877697	1.39284E-12	3.69512E-10
PPP2R5B	2.66671694	2.05689E-12	5.30089E-10
PPME1	-3.2430808	3.19299E-12	8.00022E-10

**AC104389.6**	1.51765529	3.49603E-12	8.28771E-10
**NUDT4**	1.15551401	3.50013E-12	8.28771E-10
**XPOT**	1.53566636	3.58374E-12	8.28771E-10
**ENSG00000272418**	-3.0951656	3.67526E-12	8.28771E-10
**ENSG00000285830**	1.34729276	7.92642E-12	1.74381E-09
**C12orf60**	-2.5189727	8.66542E-12	1.861E-09
**CAT**	-1.7437091	1.05152E-11	2.20575E-09
**EEF1A1**	1.4665236	1.15936E-11	2.37669E-09
**SEC62**	-1.8334015	1.77299E-11	3.55387E-09
**SMNDC1**	-1.4396419	1.85724E-11	3.64181E-09
**BNIP3L**	-1.6708491	2.29132E-11	4.39738E-09
**ENSG00000265263**	-1.6289464	3.76862E-11	7.08187E-09
**CCDC152**	-2.7812199	4.09095E-11	7.53069E-09
**ENSG00000269982**	-1.7087575	4.82142E-11	8.69785E-09
**GRAP2**	2.34138058	5.99169E-11	1.05971E-08
**IDH1**	3.22539098	9.49834E-11	1.6476E-08
**STOM**	-1.4079948	1.59641E-10	2.7169E-08
**DAPK1**	-2.3188293	1.8968E-10	3.16835E-08
**CDC27**	-1.8985189	2.21937E-10	3.63977E-08
**CNOT7**	1.23001762	5.0876E-10	8.19467E-08
**OR10Z1**	-1.1966461	5.42622E-10	8.58675E-08
**SPATA9**	-1.1034353	5.78303E-10	8.99362E-08
**SLC24A1**	-1.3000934	8.31217E-10	1.27078E-07
**TEX2**	1.50548989	9.40293E-10	1.41357E-07
**TCP11L2**	-2.0383232	9.91944E-10	1.46678E-07
**MCM5**	1.18860682	1.19326E-09	1.73599E-07
**RANBP10**	-2.8680447	1.22913E-09	1.7598E-07
**C9orf78**	-2.2511255	2.06738E-09	2.91371E-07
**CLMN**	4.48172922	2.73087E-09	3.7896E-07
**SMG1P3**	2.45177729	2.83321E-09	3.87205E-07
**HEMGN**	-1.2623691	3.11111E-09	4.18839E-07
**MBNL1-AS1**	-2.6500498	4.15617E-09	5.51303E-07
**ENSG00000175182**	-2.1569884	4.32653E-09	5.59361E-07
**ENSG00000272211**	-2.0375892	4.36818E-09	5.59361E-07
**ENSG00000279551**	-1.925053	4.40295E-09	5.59361E-07
**ENSG00000253194**	-1.5993282	4.81322E-09	6.0299E-07
**MOSPD1**	-3.7058273	5.18883E-09	6.4114E-07
**PIM1**	-1.2379079	5.41846E-09	6.60466E-07
**ENSG00000284669**	-1.6922905	6.31796E-09	7.5984E-07
**NUDT4P2**	1.54275152	7.35102E-09	8.7245E-07
**ARSG**	-1.358277	8.72563E-09	1.02068E-06
**CCDC144A**	2.23651906	8.82626E-09	1.02068E-06
**RAP1GAP**	-2.6962696	1.0246E-08	1.16987E-06
**CHST2**	-4.8768465	1.05381E-08	1.18817E-06
**COG1**	-1.0723265	1.15895E-08	1.29059E-06
**SLC19A1**	-1.4439612	1.67746E-08	1.84521E-06
**AGTR1**	-4.4141522	1.92568E-08	2.09273E-06
**HSPA5**	1.69708221	2.14206E-08	2.30016E-06
**PDE4DIP**	5.15618708	2.31852E-08	2.46036E-06
**ARID3B**	-2.3297984	2.37443E-08	2.4904E-06

**WARS1**	2.07828994	2.63523E-08	2.70421E-06
**ENSG00000269984**	-1.0989837	2.63825E-08	2.70421E-06
**ACP1**	-3.099081	3.09075E-08	3.13242E-06
**TENT5C**	-1.2195707	3.57755E-08	3.5855E-06
**GLRX5**	-1.2682948	3.70601E-08	3.67343E-06
**ENSG00000215859**	-3.2720296	6.43585E-08	6.30994E-06
**SENP6**	2.55161254	6.821E-08	6.61563E-06
**TBCEL**	-2.1755542	6.97417E-08	6.69224E-06
**PLIN2**	2.14150985	7.47679E-08	7.09901E-06
**CYREN**	-1.3391296	7.59953E-08	7.14039E-06
**ALDH6A1**	-2.9337208	9.9102E-08	9.21546E-06
**ENSG00000213742**	1.09860857	1.01315E-07	9.32511E-06
**INTS8**	-1.1800534	1.02525E-07	9.34118E-06
**ENSG00000241570**	-1.3116248	1.05543E-07	9.51996E-06
